# Task-dependent learning and memory deficits in the TgF344-AD rat model of Alzheimer’s disease: three key timepoints through middle-age in females

**DOI:** 10.1038/s41598-022-18415-1

**Published:** 2022-08-26

**Authors:** Victoria E. Bernaud, Haidyn L. Bulen, Veronica L. Peña, Stephanie V. Koebele, Steven N. Northup-Smith, Alma A. Manzo, Maria Valenzuela Sanchez, Zorana Opachich, Ashley M. Ruhland, Heather A. Bimonte-Nelson

**Affiliations:** 1 Behavioral Neuroscience and Comparative Psychology Division, Department of Psychology, Arizona Alzheimer’s Consortium, Arizona State University, 950 S. McAllister Ave., PO Box 871104, Tempe, AZ 85287 USA; 2 Arizona Alzheimer’s Consortium, 4745 N 7th St, Phoenix, AZ 85014 USA

**Keywords:** Hippocampus, Spatial memory, Working memory, Cognitive ageing, Learning and memory, Alzheimer's disease, Experimental models of disease

## Abstract

The TgF344 rat model of Alzheimer’s disease (AD) provides a comprehensive neuropathology presentation, with age-dependent development of tau tangles, amyloid-beta (A${\beta}$) plaques, neuronal loss, and increased gliosis. The behavioral trajectory of this model, particularly relating to spatial learning and memory, has yet to be fully characterized. The current experiment evaluated spatial working and reference memory performance, as well as several physiological markers of health, at 3 key age points in female TgF344-AD rats: 6-months, 9-months, and 12-months. At 6 months of age, indications of working and reference memory impairments were observed in transgenic (Tg) rats on the water radial-arm maze, a complex task that requires working and reference memory simultaneously; at 12 months old, Tg impairments were observed for two working memory measures on this task. Notably, no impairments were observed at the 9-month timepoint on this maze. For the Morris maze, a measure of spatial reference memory, Tg rats demonstrated significant impairment relative to wildtype (WT) controls at all 3 age-points. Frontal cortex, entorhinal cortex, and dorsal hippocampus were evaluated for A${\beta}$_1–42_ expression via western blot in Tg rats only. Analyses of A${\beta}$_1–42_ expression revealed age-dependent increases in all 3 regions critical to spatial learning and memory. Measures of physiological health, including heart, uterine, and body weights, revealed unique age-specific outcomes for female Tg rats, with the 9-month timepoint identified as critical for further research within the trajectory of AD-like behavior, physiology, and pathology.

## Introduction

As of 2021, the Alzheimer’s Association estimates that in the United States alone, 6.2 million individuals are living with Alzheimer’s disease (AD), and almost two-thirds of these cases are women^1^. There are currently no disease-modifying treatments to halt or attenuate the progressive neurodegeneration, memory loss, and cognitive decline that accompany this disease, making AD a tremendous public health crisis. Only after decades of pathological progression does clinical symptomology begin to present in patients with probable AD^2–4^. This lengthy preclinical stage adds to the difficulty of finding efficacious treatments, as the burden of brain changes is extensive and potentially irreparable by the time of clinical diagnosis. As such, it is pertinent that researchers characterize the trajectory of prodromal symptoms of AD, which will aid in identifying reliable measures for early diagnosis and treatment.

Transgenic rodent (Tg) models aim to reproduce pathological and behavioral characteristics of patients with AD, with the hope of probing the lengthy and largely silent prodromal stages of the disease. Cohen and colleagues generated a Tg rat model (TgF344-AD) that expresses the human amyloid precursor protein (APP) with the Swedish mutation and the mutant human presenilin-1 lacking exon 9 (PSEN1dE9)^5^. Several studies have recently demonstrated the promise of the TgF344-AD rat in modeling and enriching our understanding of the pathological and cognitive changes associated with human AD. Specifically, TgF344-AD rats develop soluble amyloid-beta (Aβ) oligomers that later aggregate into Aβ plaques, as well as endogenous hyperphosphorylated tau leading to neurofibrillary tangle-like structures that develop in an age-dependent manner^5^. Additionally, this model presents with neuroinflammation, cerebrovascular dysfunction, reduced synaptic transmission, and frank neuronal loss in AD-associated regions of the brain^6–8^.

While the temporal development of pathology in this model seems to recapitulate that seen with clinical AD, the behavioral characterizations conducted thus far using the TgF344-AD rat model have yielded conflicting results. Currently, the age at which impairments in specific memory domains emerge in the TgF344-AD model is unclear. One study demonstrated spatial reference memory deficits measured by the Morris Water Maze (MWM) in TgF344-AD rats compared to wildtype (WT) rats as early as 6 months of age^9^, whereas other studies have reported no significant spatial learning and reference memory deficits at this timepoint via the MWM or the Barnes maze^5,10^. However, at 6 months of age, Cohen and colleagues detected a trend toward impairment in TgF344-AD rats compared to WT rats in the reversal phase of the Barnes task, assessing cognitive flexibility, which is thought to depend, in part, on the frontal cortex^5^. The discordance of the studies performed to date, in addition to the early indications of memory impairment, warrant a more nuanced evaluation of spatial learning and memory at this 6-month timepoint, in which the detection of burgeoning cognitive deficits may depend on the specific type of memory being assessed as well as the paradigm used to assess it.

Further evaluations of spatial memory have been conducted for the TgF344-AD rat beyond 6 months of age. In fact, between 7 and 13 months of age, a similar pattern of discordant findings has been reported. One study evaluating spatial navigation strategy and spatial reference memory found that as early as 7–8 months of age, TgF344-AD rats showed a decrease in the directness of their swim path trajectory to the escape platform in the MWM, but not a significant impairment when analyzing swim path length, suggesting that TgF344-AD rats utilized a different navigation strategy without having overt spatial reference memory impairments at this 7–8 month timepoint^11^. In this same study, robust impairments in spatial reference memory were detected in TgF344-AD rats at 10–11 months of age. Notably, other studies have reported no significant differences in spatial reference memory between TgF344-AD and WT rats in the Barnes maze at 12–13 months of age, nor impairments in spatial or non-spatial recognition memory via two variants of the novel object recognition task in 12 month-old TgF344-AD rats^8,12^. TgF344-AD rats between 9 and 12 months of age showed impaired spatial memory, but intact non-spatial recognition memory, relative to WT rats, suggesting a dissociation of effects depending on task type^13^. Importantly, the initial characterizations of the TgF344-AD rat model by Cohen and colleagues found no observable sex differences on the tasks assessed, and consequently, a majority of the subsequent studies on this model combined male and female rats^5^. This may account for some of the discordant behavioral findings, as previous AD rodent models have displayed sex differences in cognitive function and neuropathology^14,15^, and from a clinical perspective, it is well established that females are at a greater risk of developing AD compared to males^16^. Overall, using different paradigms to assess specific memory domains, combining sexes and ages, as well as a lack of statistical power to detect nuanced memory changes, likely contribute to the lack of accordance in behavioral findings. As such, these studies prompt further investigation into the complex behavioral phenotype of the TgF344-AD rat model, particularly in females, as they are disproportionately affected by AD, and at critical age ranges for this model from young adulthood through middle-age.

The current experiment aimed to assess how spatial learning and memory changes manifest in 6, 9, and 12-month old female TgF344-AD rats, with the goal to elucidate the timeline of nuanced behavioral changes in working and reference memory domains. While many of the aforementioned studies focused on spatial navigation and long-term memory, no studies have systematically characterized spatial working memory performance as a variation of cognitive load in early adulthood. Prior work has indicated that changes in working memory may be one of the earliest cognitive symptoms associated with AD^17–19^, and working memory impairments have been shown to reliably predict the progression from mild cognitive impairment (MCI), a prodromal stage of AD, to AD dementia^20^. The present study utilized the water radial-arm maze (WRAM) to test spatial working and reference memory performance as memory load increases^21^. These assessments testing an increasing working memory demand provide a more sensitive measurement of cognitive change and a reduced likelihood of ceiling effects for animals in a preclinical stage of development. Physiological markers of health were also evaluated in female TgF344-AD rats and WT controls, and elements of brain pathology were evaluated in the Tg rats, with the goal of constructing a comprehensive picture of how the TgF344-AD rat model could translate to the presentation of AD in women. These assessments provide opportunities to discover relationships among cognitive, select brain pathology, and health related markers at the earliest stages of functional AD symptom onset.

## Methods

### Subjects

**Figure 1:**
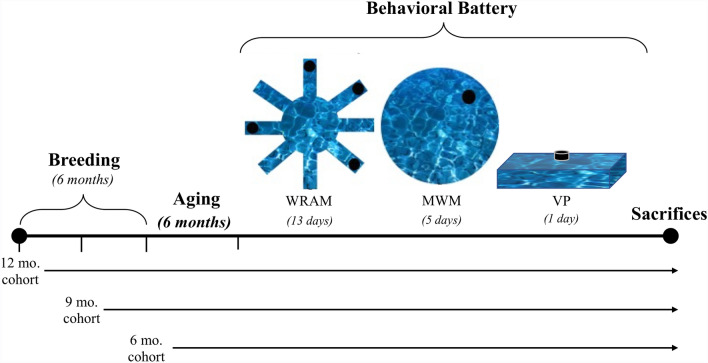
Study timeline and behavioral assessments in this novel TgF344 rat model of AD. The study timeline depicts the breeding schedule for each age cohort and the commencement of behavioral testing, including WRAM, MWM, and VP, before euthanasia (sacrifices) at the conclusion of the study.

To establish a transgenic colony at Arizona State University, a male rat carrying the APP/PSEN1dE9 transgenes and 4 female WT rats on a Fischer 344 NHSD background were obtained from the University of Southern California. These rats were used to breed subsequent generations of experimental rats within our laboratory, of which 30 WT sexually inexperienced female rats and 30 Tg sexually inexperienced female rats were used for the current study. For each genotype, rats were aged to 6, 9, and 12 months at the onset of behavioral testing, making for the following experimental groups: 6-month Tg rats (n = 10), 6-month WT rats (n = 10), 9-month Tg rats (n = 11), 9-month WT rats (n = 10), 12-month Tg rats (n = 9), and 12-month WT rats (n = 10). One subject from the 6-month Tg group was excluded from all analyses due to abnormal behavioral results and protein concentrations as analyzed via western blot. All rats were kept on a 12-h light/dark cycle (light on at 7:00 am) with ad libitum access to food and water. All experimental protocols were approved by the Arizona State University (ASU) Institutional Animal Care and Use Committee and were in accordance with guidelines from the National Institutes of Health (NIH). Furthermore, the study was completed in accordance with ARRIVE guidelines. For a brief overview of the experimental timeline, see Fig. 1.

### Animal breeding and housing

Breeding all experimental subjects involved pairing either 2 WT females with a Tg male, or 2 Tg females with a WT male within a cage. Transgenic status was confirmed via Polymerase Chain Reaction (PCR; see Genotyping section below) before selection as a breeder. A systematic breeding schedule was developed for this study to ensure that rats were the specific ages of 6, 9, and 12 months old during simultaneous behavioral evaluation. Breeding pairs were placed together in a cage a month prior to the target birth date of a litter, as the typical rat gestation period is 21 days. Litter size was between 3 to 14 pups. Before the litters were born, the male rat was removed from the cage to avoid disturbance of the dam or the litter.

To achieve the proper distribution of Tg and WT rats for each age cohort in the current study, a minimum of 10 breeding pairs were placed together. In order to breed litters with sufficient numbers of subjects, there was a broader range of ages within each cohort than initially anticipated. The 9-month cohort bred according to our optimal schedule, while the 12-month cohort breeding yielded rats closer to 11 months of age at behavioral testing, and the 6-month cohort yielded an age range of 5–6 months at behavioral testing.

On postnatal day 10, rat pups were identified as male or female by anogenital distance. At this time, a small razor blade was used to snip the tip of the tail (≤ 3 mm) of each pup. Tail snips were stored at -70ºC until later genotyping. To identify each subject, ear punches were performed directly following tail cuts. On postnatal day 21, rat pups were weaned from their dam and same-sex housed into littermate pairs. During breeding, when there was an odd number of males or females, the remaining rat was individually housed. No experimental animals were single-housed in the current study; if an experimental animal did not have a littermate, it was paired with a non-experimental animal of the same sex and age. Only females were used as experimental subjects in this study. Males were culled or used as future colony breeders.

### Genotyping

PCR was used to confirm the presence or absence of the human mutant APP transgene with tail samples collected from each rat, as previously published^22^. First, DNA from tail samples was digested using a solution with a 24:1 ratio of Sodium Chloride-Tris–EDTA buffer and proteinase K, totaling 500ul per sample. Samples were incubated overnight in a water bath heater set to 55 °C. DNA was extracted from each sample by centrifuging samples at 15,000 rpm for 10 min at 4 °C. Subsequent supernatant was added to 500ul of isopropyl alcohol. This solution was vigorously shaken to isolate the DNA before being placed into a Speedvac for 3 min to remove all liquid. The DNA strand for each sample was then resuspended in 150 µM TE buffer and placed back into the 55 °C water bath to incubate overnight. The following day, DNA samples were added to a solution with a 7:1:12.5 ratio of distilled water, 3 primers (PRP internal control, PRP reverse, and APP), and Taq buffer. Samples were then placed in a thermocycler to amplify DNA, and a gel was prepared by heating 1.8% agarose in 1× Tris–borate EDTA. For fluorescence of DNA bands, 10 μl GelRed nucleic acid gel stain was added to the gel preparation. Following use of the thermocycler, 1× green loading dye was added to each sample, and the samples were subsequently pipetted into each gel well, along with an appropriate ladder. Gels were run at 200 mV for 30 min before being placed under UV light to capture photos of the protein bands using a BioRad Gel Doc XR. A lower band (400 bp) corresponded to the presence of the APP transgene and indicated that the corresponding rat was Tg; a higher band (750 bp) indicated a WT rat. Representative genotypic results are displayed in Supplemental Fig. 1.

### Body weights and vaginal cytology

Weekly body weights were collected for 9 weeks across the duration of the experiment to determine the effects of genotype and age on body weight, as well as to assess overall animal health. One month prior to the initiation of behavioral testing, vaginal smears were completed for all subjects to monitor vaginal cytology with age and across genotype using a protocol previously established in our laboratory^23^. Vaginal smears were collected for 8 consecutive days, approximately 2 full estrous cycles, where smears were classified as 1 of 4 phases: estrus, metestrus, diestrus, and proestrus. Estrus was characterized by the presence of mostly cornified cells; metestrus by a mixture of cornified cells, round epithelial cells, needle-like cells, and leukocytes; diestrus by leukocytes with or without cornified cells; and proestrus by epithelial round cells with or without cornified cells. Characterization of each phase was completed in correspondence with previously published work^23,24^.

### Behavioral battery

Behavioral testing was carried out at the same time each morning (8:00 AM, +/− 30 min) in two separate rooms for greater efficiency. Counterbalancing was performed to ensure that there was an even distribution of ages and genotypes (i.e. 50% WT, 50% Tg) across both rooms.

### Water radial-arm maze (WRAM)

For the current experiment, the WRAM was utilized to assess spatial working and reference memory, as previously done in our laboratory^25–27^ using established protocols^28^. The WRAM involved an 8-arm apparatus filled with 18–20 °C water located in a room with robust extra-maze cues to aid in spatial navigation. Such cues included a large black and white striped rectangle (22″ by 55″) mounted to the wall in front of the maze, as well as a large black rectangle (22″ by 55″) on the adjacent wall. Other extra-maze cues included a desk, a long table, and the tester (who remained in a fixed location while the rats swam). At the ends of 4 of the 8 maze arms, platforms were hidden 2–3 cm under the water surface, further obscured by non-toxic black paint in the water. Platform locations were semi-randomized across subjects and counterbalanced across experimental groups, but were maintained across all days of testing for a given rat. At the beginning of a testing day, rats were placed into individual testing cages and brought into the testing rooms in groups of 10. At the beginning of a testing session, the rat was placed into the start arm and given 3 min to swim to 1 of the 4 hidden platforms. If the rat did not find a platform within the maximum trial time, it was led to the nearest platform, at which point the trial was complete. If the rat found the platform within the 3-min trial time, the rat was allowed to remain on the platform for a total of 15 s before being removed from the maze and placed into its heated testing cage for a 30 s inter-trial interval (ITI). During the ITI, the just-found platform was removed, and the maze was swept for odor cues. After the ITI, the rat was placed back into the start arm of the maze and given 3 min to swim and navigate to a remaining platform. Trials continued in this manner until all 4 platforms were located, allowing for an incremental increase in working memory load across testing trials, as rats had to remember not to re-visit arms where platforms were located earlier within a day. Upon completion of the 4th trial, the rat was returned to its testing cage, and behavioral testing for the next rat was initiated. Testing was completed across 2 testing rooms simultaneously for all experimental subjects. Rats were tested in this manner for a total of 12 baseline days of testing. On the 13th day of WRAM testing, a 6-h delay was implemented between trials 2 and 3 to evaluate delayed memory retention.

Performance on this task was evaluated via errors, or the number of entries a rat made into an arm that did not contain a platform prior to locating a platform on a given trial. Errors were further categorized into 3 types: working memory correct (WMC) errors, reference memory (RM) errors, and working memory incorrect (WMI) errors. WMC errors were entries into arms that previously contained a platform, indicating the rat remembered a correct platform location but failed to update their memory by recognizing they had already found that platform during that day of testing. RM errors were first entries into a non-platformed arm for a given testing day, reflecting a failure in long-term memory. WMI errors were each subsequent entry into a non-platformed reference memory arm for a given testing day, demonstrating the rat failed to update they already visited that arm and were not rewarded.

### Morris water maze (MWM)

Upon completion of the WRAM, rats commenced MWM testing the following day to evaluate spatial reference memory, as done previously in our laboratory^27,29^ using published protocols^30^. The MWM consisted of a large circular pool filled with 18–20 °C water made opaque with non-toxic black paint, surrounded by robust extra-maze cues to aid in spatial navigation. These cues included a large black and white striped rectangle (22″ by 42″) mounted to the wall in front of the maze, as well as a large black rectangle (22″ by 28″) with a smaller white rectangle inside it (11″ by 14″) on the opposing wall. In addition to these cues, a computer desk, a long table, and the tester (who remained in a fixed location while the rats swam) served as additional extra-maze cues. In the northeast quadrant of the maze, a platform was submerged 2–3 cm underneath the water, where it remained for the duration of baseline testing. Across all baseline testing trials, rats were dropped off semi-randomly from 4 different locations (labeled N, S, E, W) around the maze and given 1 min to swim to the platform. If the rat did not find the platform within the 1-min trial time, it was led to the platform and the trial was terminated. Once on the platform, the rat remained there for 15 s before being placed back into its testing cage. The maze was then swept for odor cues and debris before the next rat was placed into the maze. The ITI for this task was approximately 10–15 min, as all rats completed one trial before moving on to the next trial. Rats completed 4 trials per day for 5 testing days. On the 5th testing day, a final 5th trial was added as a probe trial, where the platform was removed from the maze. The rat was allowed to swim for 1 min, permitting evaluation of spatial localization to the platform.

MWM performance was assessed by swim distance and swim latency to the platform. To evaluate these measures, a video camera and computer equipped with Ethovision 6 (Noldus Instruments, Wageningen, The Netherlands) were used to record trials. For the probe trial, percent swim distance in the quadrant that previously contained the platform (Target Quadrant) and percent swim distance in the quadrant opposite the platform (Opposite Quadrant) were recorded for each rat.

### Visible platform (VP)

Following the MWM task, rats were evaluated on the single day VP task, which assesses visual and motor acuity, as well as ability to perform the procedural components necessary to solve a water-escape task. The VP task followed established protocols^31^ that have been used in prior work from our laboratory^26^. This task consisted of a 100 cm × 60 cm tub filled with clear 18–20 °C water surrounded by a large curtain to obscure any extra-maze cues. The back of the tub contained a black platform, placed 2–3 cm above the water’s surface. The platform was semi-randomly moved to 3 different locations across all 6 testing trials. At the beginning of the testing day, a rat was dropped off at the starting location and given 90 s to reach the platform. If the rat did not locate the platform in the given trial time, the rat was led to the platform, and the trial ended. If a rat was unable to locate the platform within this time on multiple trials, we considered this subject for exclusion from the data analysis on all behavioral tasks, as this could indicate an impaired ability to complete a water-escape based task. Upon locating the platform, the rat remained there for 15 s before being removed and placed into its heated testing cage. The maze was then swept for odor cues and debris before the next rat began its trial. Testing continued in this fashion, with an average ITI of 5–8 min for all 6 testing trials. Swim latency to platform was recorded for VP performance.

### Tissue collection

After conclusion of the behavioral battery, all rats were euthanized to collect tissues for further processing. At euthanasia, rat ages were approximately 7 months (termed the 6-month groups), 10 months (termed the 9-month groups), and 13 months (termed the 12-month groups). Rats were deeply anesthetized with isoflurane before blood was collected via cardiocentesis. Hearts were then weighed (wet weight). Brains were extracted for rapid dissection, as done in previous work from our laboratory^32^. Brain dissections were performed using Eagle Medium kept on ice; dissected regions including the right Frontal Cortex (FC; Plates 5–14), right Entorhinal Cortex (EC; Plates 39–42), and right Dorsal Hippocampus (dHPC; Plates 33–35) were collected as previously described^33^. Upon collection of each region, tissues were placed in individual pre-weighed microcentrifuge tubes, weighed, and stored at – 70 °C until further analysis. Left and right ovaries and uterine horns were also collected from each subject, trimmed of any abdominal fat, and weighed (wet weight).

### Western blot protein analysis

Samples of FC, EC, and dHPC brain regions from the right hemisphere of each Tg subject (N = 30) were processed for relative expression of A$\beta$_1–42_ via western blot protein analysis. Tissue samples kept at − 70 °C were first suspended in a 1:25 weight-to-volume ratio of RIPA buffer solution (150 mM NaCl, 1% Triton X-100, 0.1% SDS, 0.5% sodium deoxycholate, 50 mM Tris HCl), protease inhibitor (Millipore-Sigma) and phosphatase inhibitor (Millipore-Sigma), with sample tubes kept thereafter over ice. Tissues were homogenized using probe sonication (Ultrasonic Processor, Cole Parmer, IL, USA), and homogenate was centrifuged at 10,000 rpm for 10 min at 4 °C. The resulting supernatant was collected and aliquoted to avoid freeze–thaw cycles for the samples. Aliquots were stored at − 70 °C. The protein concentration of each sample was determined using a bicinchoninic acid protein assay (BCA; Thermo-Fischer Scientific, Pittsburgh, PA, USA), where samples were run in duplicate and their concentration values averaged, with no higher coefficient of variation between the duplicate samples than 11%. One sample from the dHPC of a subject in the 9-month Tg group had a protein concentration that exceeded the level of detection for this assay, resulting in the exclusion of this sample from western blot analysis.

Samples of differing ages were counterbalanced across each gel. Each gel also contained a positive and negative control from aged subjects in the colony that were confirmed to be Tg and WT, respectively. The NuPAGE PowerEase electrophoresis system was used for tissue processing. Samples for a given region were loaded in a 4–12% NuPAGE Bis–Tris gel in an XCell SureLock Mini-Cell (Invitrogen, Carlsbad, CA, USA), each with 10ug protein and run with MES running buffer. After protein separation, transfer was completed using an Immobilon polyvinylidene difluoride membrane. Following protein transfer, the membrane was incubated in 5% nonfat milk for 1 h at room temperature before being washed with 1× TBST. The membrane was then incubated at 4 °C overnight in the primary antibody for A$\beta$_1–42_ (1:1,000; Cell Signaling #12843S) and the loading control beta-actin (1:20,000; Cell Signaling #4970S) in 5% milk. The following day, the membrane was washed with 1× TBST before incubation for 1 h at room temperature in the secondary antibody anti-rabbit HRP (1:500; Cell Signaling #7074). The membrane was washed with 1× TBST and immunoreactivity was visualized with enhanced chemiluminescence (Lumiglo and peroxide solutions, Cell Signaling #7003S) and developed on a Konica SRX-101A Film Processor (Tokyo, Japan). Densitometry analyses were completed for resulting films using ImageJ software based upon previously established protocols^34^. A$\beta$_1–42_ expression was normalized to the expression of the loading control beta-actin for each gel. A total of 9 gels were run across all 3 brain regions.

### Statistical analyses

Statistical analyses were completed using StatView software. All behavioral data, with the exception of the WRAM delay and MWM probe trial, were analyzed using a series of planned comparisons which investigated potential effects of Genotype for each age (i.e., 6-month Tg vs. WT, 9-month Tg vs. WT, 12-month Tg vs. WT). This allowed us to address whether there were behavioral or neurobiological differences across genotype for each specific age cohort. All analyses were two-tailed, with an alpha level set at 0.05. Results were considered marginal where the *p* value was between 0.05 and 0.10.

For WRAM data, repeated measures ANOVAs were utilized for each set of planned comparisons within an age cohort, with Genotype as the independent variable, Errors as the dependent variable (with separate analyses for WMC, RM, and WMI errors), and Days and Trials as the repeated measures. Based on prior publications from our laboratory that have demonstrated differences in baseline testing days corresponding to performance in task acquisition and maintenance^25,27,35,36^, WRAM data were analyzed following the set of planned comparisons outlined above in 2 separate blocks: the Acquisition Phase (Days 2–7) and the Asymptotic Phase (Days 8–12). These 2 blocks were also chosen based on the WRAM learning curve across all days of testing for these rats, whereby all rats had a steeper decrease in making errors from Days 2–7 while acquiring the task rules, with performance plateauing from Days 8–12, maintaining task performance. For data collected during the WRAM delay, each experimental group was analyzed individually with a repeated measures ANOVA, where WMC errors served as the dependent variable, and Trial 3 performance on the last baseline day of testing (Day 12) versus Trial 3 performance directly following the delay (Day 13) served as the repeated measure.

MWM data were analyzed using a repeated measures ANOVA in the same set of planned comparisons as previously described, where Genotype was the independent variable, Swim Distance (cm) or Swim Latency (s) to the Platform was the dependent variable, and Trials within Days was the repeated measure. The probe trial was analyzed for each experimental group using a repeated measures ANOVA, where percent swim distance in the Target (NE) or Opposite (SW) Quadrant served as the within-subjects dependent variable.

For the VP task, repeated measures ANOVA were run for the same set of planned comparisons, with Genotype as the independent variable, Latency (s) to the Platform as the dependent variable, and Trials as the repeated measure.

Weekly body weight data were analyzed using repeated measures ANOVA with the same set of planned comparisons as described above, where Genotype was the independent variable, Body Weight was the dependent variable, and Weeks was the repeated measure. Other organ weights collected at the end of the experiment were analyzed using ANOVA with the same set of planned comparisons, where Genotype served as the independent variable and Wet Weight or the ratio of Wet Weight to Body Weight as the dependent variable.

Data from all Tg subjects for western blot protein analyses were analyzed via ANOVA, with Age as the independent variable and normalized relative expression of A$\beta$_1–42_ as the dependent variable, and individual analyses were run for each brain region. With a significant effect of Age, post hoc analyses were performed using Fisher protected least significant difference (PLSD). One subject in the 6-month Tg group had levels of A$\beta$_1–42_ in several brain regions that far exceeded that of the group; this subject was subsequently removed from all behavioral and brain analyses, as mentioned previously.

To determine putative relationships between behavioral, physiological, and neurobiological outcomes within Tg rats, a series of Pearson r correlations were completed with Fisher’s tests of significance. Specifically, WMC and WMI errors summed across trials and averaged across days for each block of WRAM testing were correlated with A$\beta$_1–42_ expression in the FC, dHPC, and EC to determine whether there was a relationship between working memory performance and amyloid-beta expression in these brain regions. MWM Swim Latency and Distance summed across trials and across days were correlated with A$\beta$_1–42_ expression in the FC, dHPC, and EC to determine if there was a relationship between reference memory performance and A$\beta$_1–42_ expression in these regions. Additionally, body weight at the end of the experiment was correlated with A$\beta$_1–42_ expression in the FC, dHPC, and EC to determine whether a relationship existed between physiological health and amyloid-beta protein expression. These correlations were performed separately for each Tg experimental group (i.e., intraclass correlations). Given the exploratory nature of these correlations, corrections for multiple comparisons were not performed; the threshold of significance was set to *p* < 0.01 for all correlations.

## Results

### Vaginal cytology

Vaginal smears performed across 8 consecutive days resulted in observations of age-typical estrous cyclicity, with most rats exhibiting normal cycles 4–5 days in length. Generally, the number of rats with irregular estrous cyclicity (e.g., prolonged cycles, persistent estrus/diestrus) increased with age: only 1/10 WT subject in the 6-month cohort was observed to have an irregular cycle, where 10 (5/10 = WT, 5/11 = Tg) rats in the 9-month cohort and 7 (5/10 = WT, 2/9 = Tg) rats in the 12-month cohort had irregular estrous cyclicity. These irregular estrous cycles did not appear to differ by genotype. This would indicate that WT and Tg rats had normally functioning ovaries, where there were indications of irregular cycles with increasing age as commonly observed in prior research^23,37^.

### Behavioral battery

#### WRAM

##### Baseline testing

**Figure 2:**
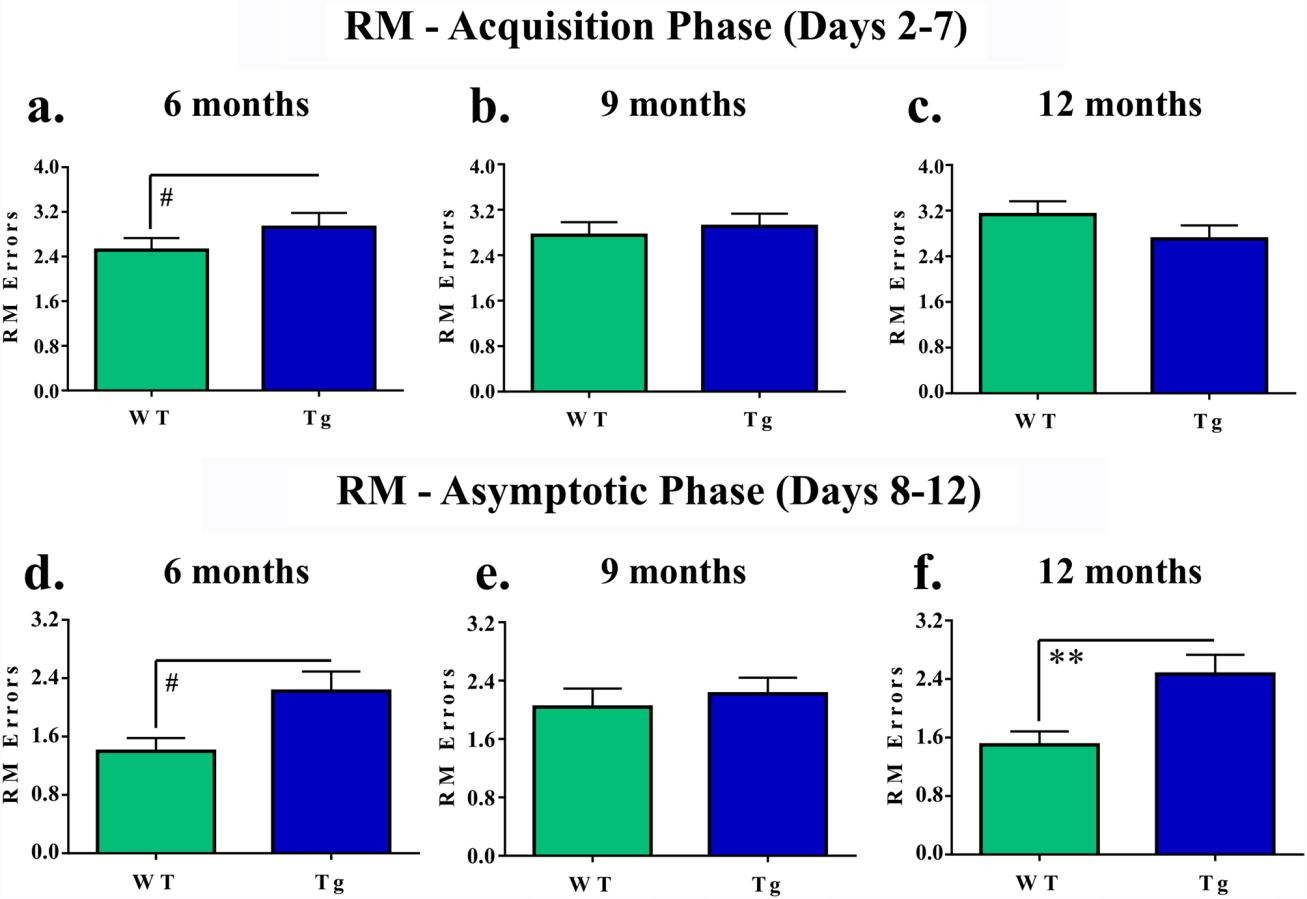
Distinct patterns of reference memory deficits in TgF344-AD rats as evaluated on the WRAM. (**a**–**c**) During task acquisition, 6-month Tg rats tended to make more errors than their WT counterparts; there were no effects for RM errors in the 9-month or 12-month groups. (**d**–**f**) During task maintenance, 6-month Tg rats tended to make more errors than their WT counterparts. While there were no differences amongst the 9-month groups, at the 12-month timepoint, Tg rats made significantly more errors than WT rats. #*p* < 0.10, **p < 0.01.

Analysis of performance during the Acquisition Phase captures learning of the WRAM task, and analysis of performance during the Asymptotic Phase captures task memory maintenance. For RM errors during the Acquisition Phase, there was a marginal main effect of Genotype for the 6-month groups [*F*_(1,17)_ = 3.65, *p* = 0.0732, whereby Tg rats tended to make more RM errors compared to WT rats (Fig. 2a). There were no other effects for RM errors for the 9 or 12-month groups for this initial phase of testing (Fig. 2b, c), nor were there main effects or interactions with Genotype for WMC or WMI errors for any age-specific planned comparisons.

For RM errors during the Asymptotic Phase, there was a marginal main effect of Genotype for the 6-month planned comparison [*F*_(1,17)_ = 4.27, *p* = 0.0544], such that Tg rats made marginally more RM errors than WT rats (Fig. 2d). Although there were no RM effects for the 9-month groups (Fig. 2e), for the 12-month planned comparison, there was a main effect of Genotype for RM errors [*F*_(1,17)_ = 14.87, *p* = 0.0013], with Tg rats making more errors than WT rats in this oldest cohort, demonstrating a reference memory impairment among Tg rats (Fig. 2f).

**Figure 3:**
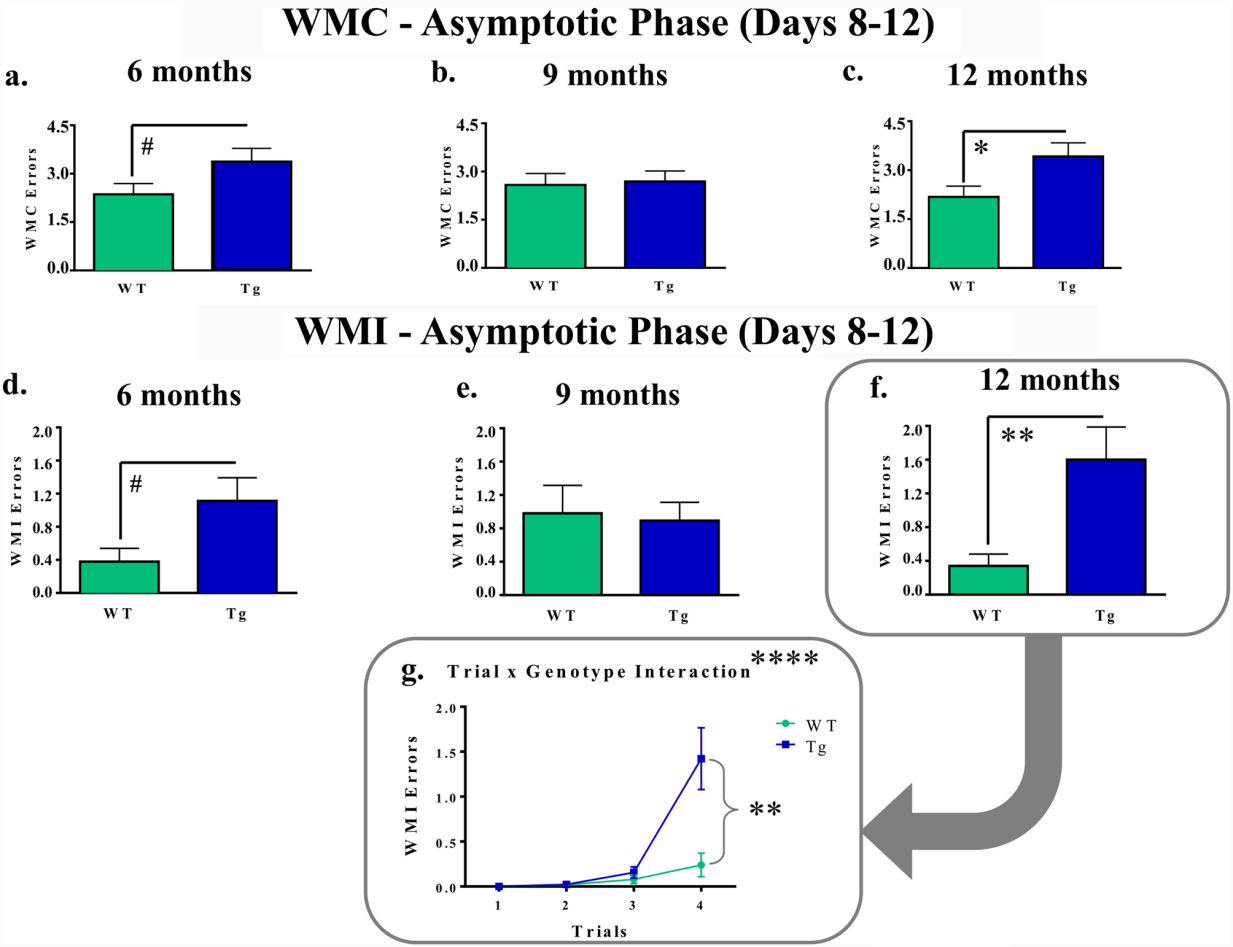
Distinct patterns of working memory deficits in TgF344-AD rats as evaluated on the WRAM. (**a**–**c**) During the Asymptotic Phase of WRAM testing, 6-month Tg rats made marginally more WMC errors than WT rats. Tg rats at the 12-month timepoint made significantly more WMC errors than WT counterparts, indicating working memory impairment. (**d**–**f**) Likewise, in the Asymptotic Phase of the WRAM, Tg rats in the 6-month cohort made marginally more WMI errors than WT rats of the same age. At the 12-month timepoint, Tg rats overall made significantly more WMI errors than WT rats. (**g**) Following up on a Trial × Treatment interaction, a genotype effect was found during the highest working memory load trial, with Tg rats making more WMI errors than WT rats at 12-months of age. #*p* < 0.10, *p < 0.05, **p < 0.01, ****p < 0.0001.

During the Asymptotic Phase of WRAM testing, for WMC errors, there was a marginal effect of Genotype for the 6-month cohort [*F*_(1,17)_ = 3.09, *p* = 0.0967], where Tg rats tended to make more errors than their WT counterparts (Fig. 3a). While there were no significant Genotype effects for the 9-month cohort (Fig. 3b), for the 12-month cohort, there was an effect of Genotype [*F*_(1,17)_ = 4.90, *p* = 0.0408], where Tg rats made more WMC errors than WT rats (Fig. 3c). There were no Trial × Genotype interactions for WMC errors across any of the age-specific planned comparisons.

For WMI errors during the Asymptotic Phase, planned comparisons of the 6-month groups yielded a marginal effect of Genotype [*F*_(1,17)_ = 3.27, *p* = 0.0883], whereby Tg rats tended to make more errors than WT rats (Fig. 3d). No effects of Genotype were present for the 9-month groups (Fig. 3e). Similar to WMC errors, there was an effect of Genotype for WMI errors during the Asymptotic Phase for the 12-month groups [*F*_(1,17)_ = 8.50, *p* = 0.0096], with Tg rats making overall more WMI errors than WT rats (Fig. 3f). Furthermore, there was a significant Trial × Genotype interaction for this planned comparison [*F*_(3,51)_ = 10.02, *p* < 0.0001]. When the highest working memory load trial, Trial 4, was probed separately, there was a Genotype effect [*F*_(1,17)_ = 10.18, *p* = 0.0054], where 12-month Tg rats made more errors compared to 12-month WT rats (Fig. 3g).

##### Delayed memory retention

**Figure 4:**
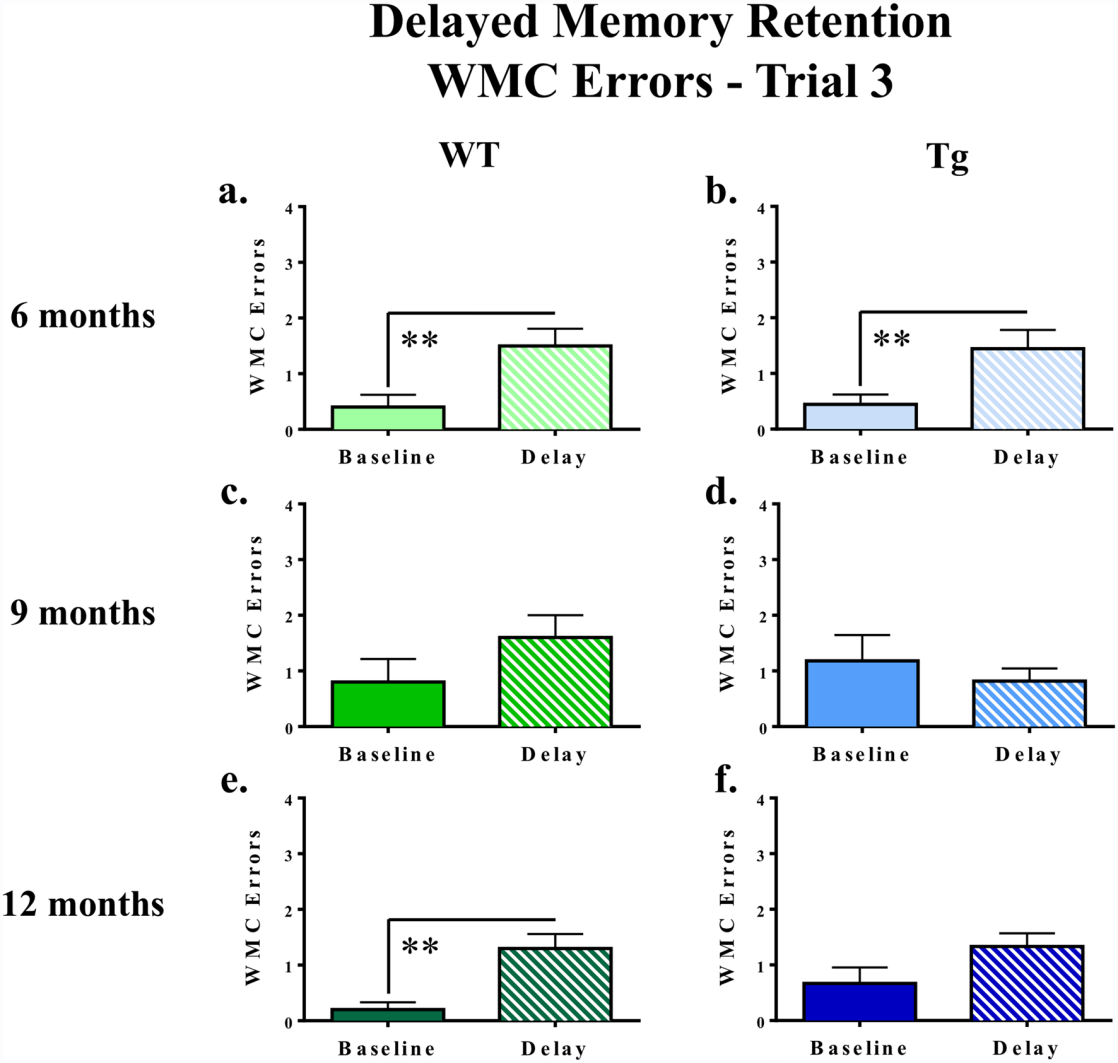
Assessments of delayed memory retention on the WRAM. Following implementation of a 6-h delay on Day 13 of the WRAM, analyses of performance in WMC errors for Trial 3 on Day 12 (Baseline) and Day 13 (Delay) revealed a significant effect of the delay for rats in the 6-month Tg group, the 6-month WT group, and the 12-month WT group. **p < 0.01.

To evaluate delayed memory retention, a 6-h delay was implemented between Trials 2 and 3 on the final day of WRAM testing. Performance was evaluated by comparing WMC errors made on Trial 3 the final day of baseline testing (Day 12) to WMC errors made on Trial 3 directly following the delay (Day 13). For 6-month WT rats [*F*_(1,9)_ = 12.24, *p* = 0.0067] and 6-month Tg rats [*F*_(1,8)_ = 12.00, *p* = 0.0085], there was a main effect of Delay for WMC errors, where rats made more errors following the implementation of a delay (Fig. 4a and b, respectively). There were no effects of the delay for the 9-month rats (Fig. 4c and d). There was a main effect of Delay for WMC errors for the 12-month WT group [*F*_(1,9)_ = 15.78, *p* = 0.0032], where WT rats made more errors directly following the delay (Fig. 4e); there was no delay effect for 12-month Tg rats (Fig. 4f).

#### MWM

**Figure 5:**
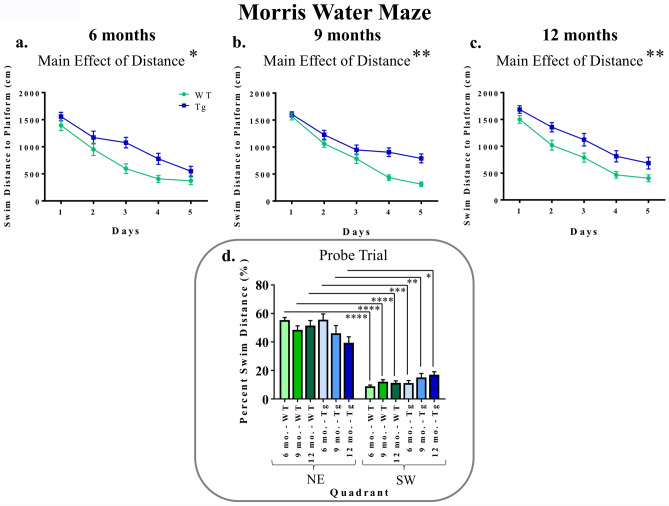
Reference memory performance as evaluated on the MWM: consistent deficits in TgF344-AD rats across each age for swim distance to the platform. (**a**–**c**) When evaluating swim distance to platform (cm) for each age-point across Days 1–5, there was a significant effect of Genotype, whereby Tg rats swam greater distances to locate the hidden platform than WT rats, indicating spatial reference memory impairment. *p < 0.05, **p < 0.01. (**d**) For the probe trial, analyses of percent of total swim distance in the target quadrant (NE) as compared to the opposite quadrant (SW) for each group showed that all groups were successful in spatially localizing the platform by swimming a greater proportion of their total distance in the NE as opposed to the SW. *p < 0.05, **p < 0.01, ***p < 0.001, ****p < 0.0001.

**Figure 6:**
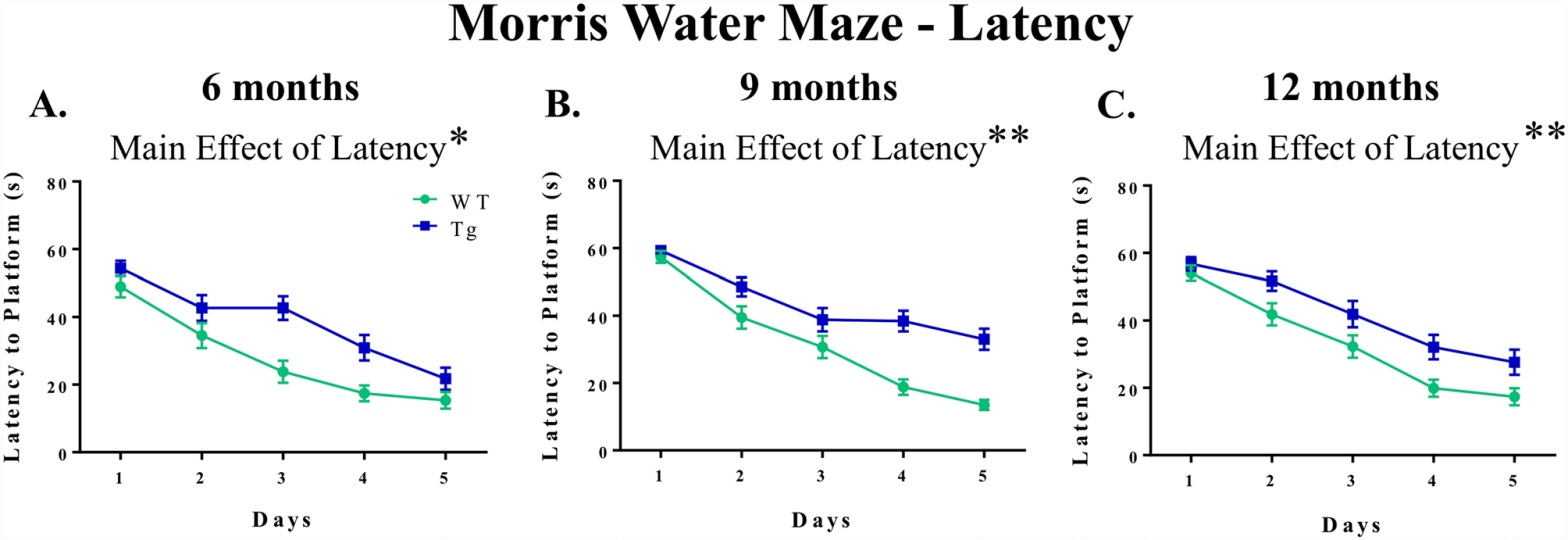
Reference memory performance as evaluated on the MWM: consistent deficits in TgF344-AD rats across each age for latency to the platform. (**a**–**c**) When evaluating latency to the platform (s) for each age-point across Days 1–5, there was a significant effect of Genotype, whereby Tg rats swam longer to locate the hidden platform than WT rats, indicating spatial reference memory impairment. *p < 0.05, **p < 0.01.

MWM data were analyzed across all 5 days of baseline testing to evaluate reference memory performance. For each set of age-specific planned comparisons, when evaluating swim distance to the platform for Days 1–5, there was a main effect of Genotype [6-month groups: *F*_(1,17)_ = 5.63, *p* = 0.0297; 9-month groups: *F*_(1,19)_ = 9.41, *p* = 0.0063; 12-month groups: *F*_(1,17)_ = 11.37, *p* = 0.0036] (Fig. 5a–c). Likewise, when evaluating swim latency to the platform collapsed across all days and trials of the MWM, there was an effect of Genotype for each set of planned comparisons [6-month groups: *F*_(1,17)_ = 7.00, *p* = 0.0170; 9-month groups: *F*_(1,19)_ = 11.31, *p* = 0.0033; 12-month groups: *F*_(1,17)_ = 7.76, *p* = 0.0127] (Fig. 6a–c). These collective data indicate a transgenic-induced reference memory impairment, as Tg rats at each age had greater distances and longer latencies to reach the platform than respective WT rats.

For each experimental group, when evaluating percent total swim distance in the Target (NE) Quadrant versus the Opposite (SW) Quadrant during the probe trial, there was a main effect of Quadrant [6-month Tg: *F*_(1,8)_ = 46.98, *p* = 0.0001; 6-month WT: *F*_(1,9)_ = 174.68, *p* < 0.0001; 9-month Tg: *F*_(1,10)_ = 11.91, *p* = 0.0062; 9-month WT: *F*_(1,9)_ = 52.79, *p* < 0.0001; 12-month Tg: *F*_(1,8)_ = 9.53, *p* = 0.0150; 12-month WT: *F*_(1,9)_ = 45.46, *p* < 0.0001] (Fig. 5d), where each group swam a greater percent of the total distance in the NE quadrant, where the platform was previously located, compared to the opposite SW quadrant. This indicates that each group spatially localized to the platform by the end of testing.

#### VP

To ensure that subjects had the visual and motor acuity necessary to complete the procedural components of a water escape task, VP data were analyzed for all experimental groups according to our set of planned comparisons. Across all 6 trials, when evaluating latency to the platform, there was a main effect of Trial for each set of planned comparisons [6-month groups: *F*_(1,17)_ = 5.85, *p* = 0.0001; 9-month groups: *F*_(1,19)_ = 4.98, *p* = 0.0004; 12-month groups: *F*_(1,17)_ = 5.28, *p* = 0.0003] (Supplemental Fig. 2a–c), with latency decreasing across testing trials; there was no effect of Genotype for any of the planned comparisons. For the 12-month groups, there was a significant Trial × Genotype interaction [*F*_(5,85)_ = 2.98, *p* = 0.0158]. This interaction appeared to be dependent upon Trial 1, where on average, Tg rats had longer swim latencies than their WT counterparts; this is likely due to exploratory behavior, and not the result of an inability to complete the task. Overall, these effects suggest the rats were capable of performing a water-escape task, and that age and genotype did not significantly alter this ability.

### Physiological markers of health

#### Body weight

**Figure 7:**
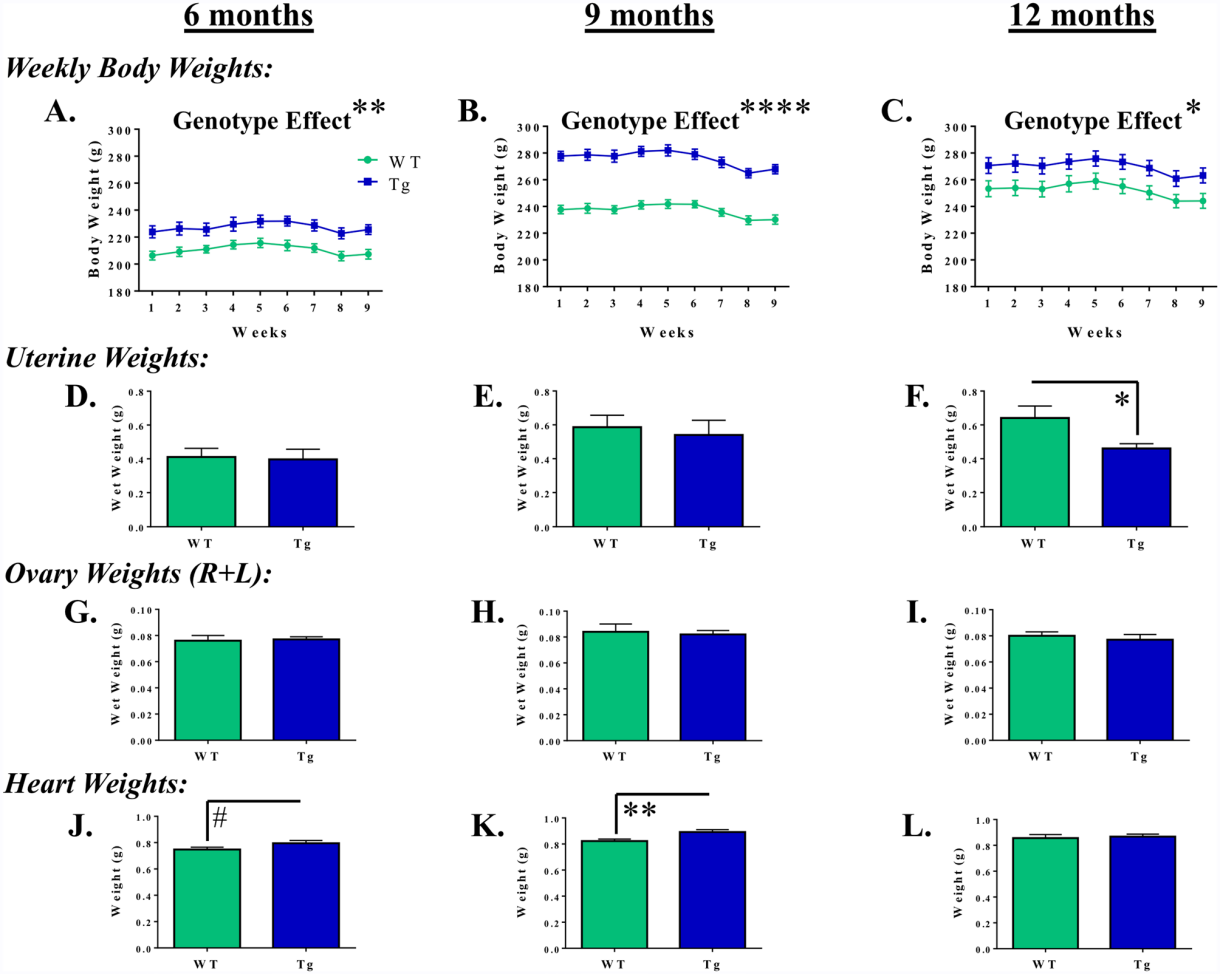
Distinct age-dependent physiological profiles in the TgF344-AD rat. (**a**–**c**) Weekly body weights collected across the course of the study indicate that, regardless of age, Tg rats weighed more than their WT counterparts. *p < 0.05, **p < 0.01, ****p < 0.0001. (**d**–**f**) Analyses of uterine weights collected at sacrifice demonstrated that, uniquely at the 12-month timepoint, Tg rats had reduced uterine weights as compared to WT controls. *p < 0.05. (**g**–**i**) Analyses of ovary weight (R + L) at sacrifice revealed no effect of Genotype at any age-point. (**j**–**l**) Analyses of heart weight demonstrated a marginal effect of Genotype at 6 months and a significant effect at 9 months, where Tg hearts weighed more than WT hearts. #*p* < 0.10, **p < 0.01.

Across the duration of the experiment, for each set of planned comparisons, there was a main effect of Genotype for body weights [6-month groups: *F*_(1,17)_ = 10.66, *p* = 0.0046; 9-month groups: *F*_(1,19)_ = 65.20, *p* < 0.0001; 12-month groups: *F*_(1,17)_ = 5.04, *p* = 0.0384] (Fig. 7a–c), where Tg rats weighed more than their WT counterparts across all ages assessed. For each age, there was an effect of Week [6-month: *F*_(8,136)_ = 13.06, *p* < 0.0001; 9-month: *F*_(8,152)_ = 37.88, *p* < 0.0001; 12-month: *F*_(8,136)_ = 23.19, *p* < 0.0001], with weights increasing until the initiation of water maze testing, and thereafter the weights began to decrease (likely due to maze-related physical activity).

#### Uterine and ovary weights

For the uterine tissue collected at euthanasia, there was no effect of Genotype on wet weight for the 6-month and 9-month timepoint planned comparisons; however, for the 12-month timepoint, there was an effect of Genotype, where WT rats had heavier uteri than their Tg counterparts [*F*_(1,17)_ = 5.08, *p* = 0.0377] (Fig. 7d–f). When evaluating ovary weights collected at euthanasia, there were no effects of Genotype across any of the age-specific planned comparisons (Fig. 7g–i).

#### Heart weights

Hearts weights collected at euthanasia revealed a marginal Genotype effect for the 6-month timepoint [*F*_(1,17)_ = 3.03, *p* = 0.0998] and a significant Genotype effect for the 9-month timepoint [*F*_(1,19)_ = 8.36, *p* = 0.0094], with Tg hearts weighing more than WT hearts (Fig. 7j–k). At 12-months, there was no effect of Genotype on heart weight (Fig. 7l). When evaluating heart weight with respect to body weight, as has previously been done^38^, the pattern of results changed: at the 9-month timepoint, there was a significant effect of Genotype [*F*_(1,19)_ = 6.94, *p* = 0.0163], where the WT heart-to-body weight ratio was greater than that of the Tg rats (data not shown). There were no other significant Genotype effects across the other age-points.

### Western blot protein analysis

**Figure 8:**
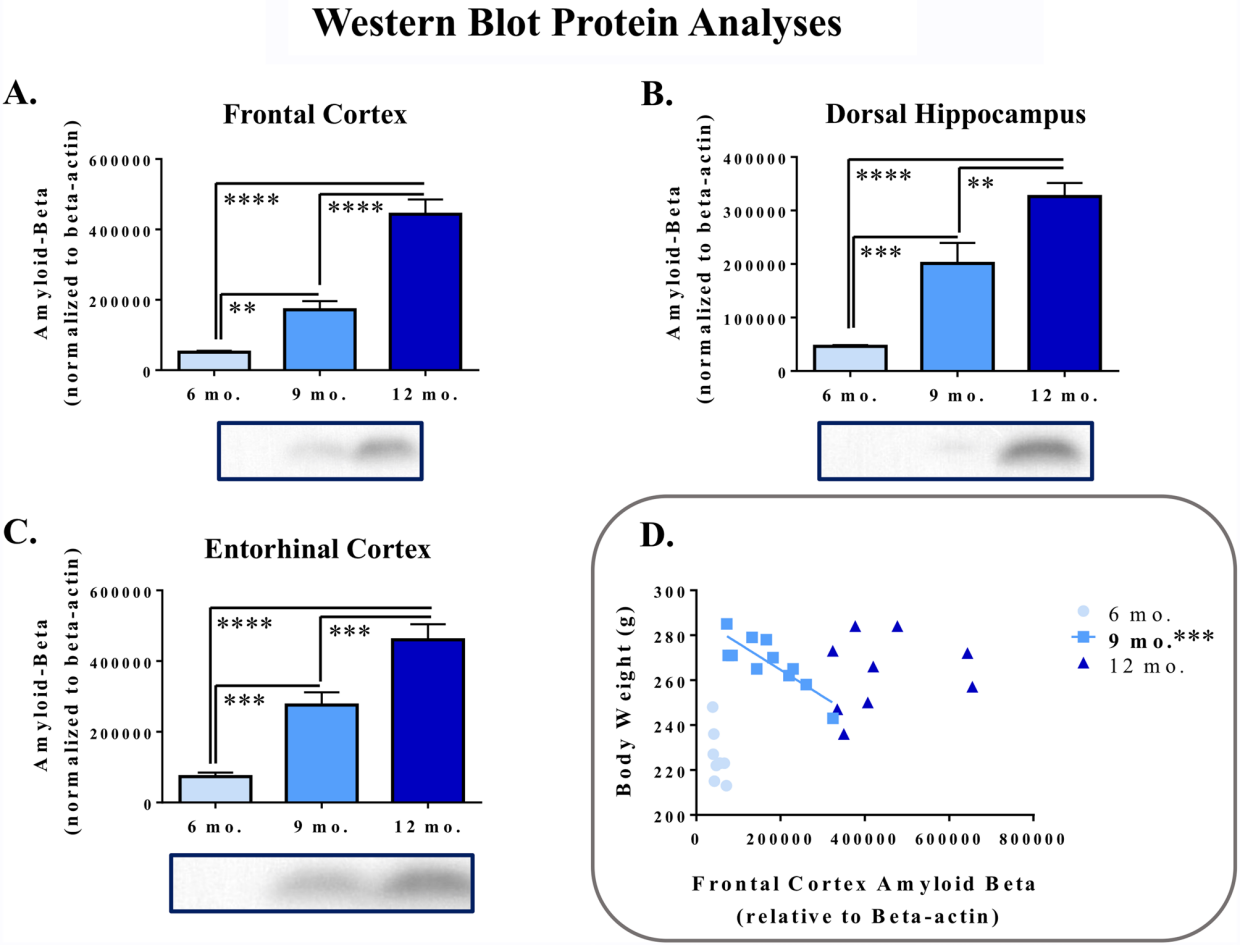
Relative expression of A$\beta$_1–42_ in frontal cortex, dorsal hippocampus, and entorhinal cortex follows an age-dependent trajectory, and relationships with body weight are unique to the 9-month old TgF344-AD rat. Western blot protein analyses demonstrated increasing relative normalized A$\beta$_1–42_ expression with increasing age in (**a**) frontal cortex, (**b**) dorsal hippocampus, and (**c**) entorhinal cortex. Cropped representative blots with bands of A$\beta$_1–42_ expression (4 kDa) and beta-actin expression (45 kDa) for each age point are demonstrated below each graph for a given brain region (full-length blots are found in Supplemental Fig. 3a–f). Post hoc comparisons are represented in this figure—main effects of age are listed within the “Results” section. **p < 0.01, ***p < 0.001, ****p < 0.0001. (**d**) For the 9-month timepoint, a unique negative correlation exists between body weight and relative normalized A$\beta$_1–42_ expression in the frontal cortex, such that increasing A$\beta$_1–42_ expression was associated with a reduction in body weight. ***p < 0.001.

Western blot protein analysis for the FC revealed a main effect of Age for A$\beta$_1–42_ expression, normalized to the loading control beta-actin [*F*_(2,26)_ = 48.84, *p* < 0.0001], where relative A$\beta$_1–42_ expression increased with age (Fig. 8a). Likewise, there was a main effect of Age for relative A$\beta$_1–42_ expression in the dHPC [*F*_(2,25)_ = 24.65, *p* < 0.0001] (Fig. 8b) as well as in the EC [*F*_(2,26)_ = 29.80, *p* < 0.0001] (Fig. 8c), in which greater expression was observed with increasing age. Cropped images of representative bands from these blots are shown in Fig. 8 to best demonstrate target bands; representative images of the corresponding full-length blots are found in Supplemental Fig. 3. There were no effects of Age for the loading control beta-actin across any brain region. These results indicate that, across these timepoints, A$\beta$ expression is increasing within brain regions critical to learning and memory outcomes for female Tg subjects.

### Correlations

Correlations between western blot and behavioral outcomes were performed for each experimental group. Specifically, correlations were run for A$\beta$_1–42_ expression in the FC, dHPC, and EC with WMC and WMI errors across the Acquisition and Asymptotic Phases of WRAM testing as well as with Swim Latency and Distance for MWM. No significant correlations were found for these variables. In addition, correlation analyses were run for body weights at the end of the experiment and A$\beta$_1–42_ expression in the FC, dHPC, and EC to evaluate potential relationships between physiological and neurobiological outcomes in Tg rats. For the 9-month Tg group, there was a significant negative correlation between normalized A$\beta$_1–42_ expression in the FC and body weight (*r* = − 0.84, *p* = 0.0006), such that with increasing A$\beta$ expression, there was a corresponding decrease in body weight **(**Fig. 8d). There were no other significant correlations across any of the other experimental groups, or in any other brain region.

## Discussion

The present study examined the manifestation of spatial learning and memory impairments as well as A$\beta$_1–42_ protein expression in female TgF344-AD rats across 3 different age-points. In doing so, we aimed to better characterize the cognitive and neurobiological trajectory of this relatively novel transgenic rat model of AD, while focusing on key timepoints critical to female aging, as clinically, females are more susceptible to developing AD. Our results demonstrated that, while the trajectory of AD-like pathology followed a linear path, the behavioral trajectory of these rats was more complex, with consistent indications of early working memory impairment at the 6-month timepoint before a robust impairment at the 12-month timepoint. Furthermore, physiological characterizations revealed unique transgenic, age-dependent outcomes that are promising in providing the field with avenues for further research into the full characterization of AD.

Behavioral evaluations on the WRAM revealed that, across reference memory and working memory performance, early indications of learning and memory impairments in the TgF344-AD rat were present in the 6-month cohort. These repeated patterns of marginal transgenic impairment across each memory error type, along with prior work demonstrating marginal impairment of reversal learning in 6-month old TgF344-AD rats on the Barnes maze^5^, form a behavioral profile that would suggest potential significant impairment if the working memory load was increased. Prior work in young, non-transgenic rats has demonstrated a lack of significant treatment- or age- related impairment when the working memory load is low, with more profound effects observed with a higher working memory load^39–41^. The early indications of memory impairment within the 6-month cohort observed here are clinically relevant, as researchers have put forth efforts to find tasks that might be able to detect AD-related memory impairments at earlier timepoints^42^ and that can distinguish AD from other forms of dementia^43^. Prior work has indicated that there are prodromal changes in working memory functioning with AD^17–19^, leading researchers to conclude that spatial navigation tasks, similar to those used in the current experiment, should be included in early clinical evaluations to detect early manifestations in functional decline with AD^44–46^. Future work should better characterize these early indications of working memory impairment, as they may aid in detection of incipient cognitive decline and correspond to key points of pathological progression in the TgF344-AD rat model. Such relationships could hold therapeutic potential, as current treatments for AD are only initiated after the disease has progressed profoundly in its pathological and cognitive trajectory^47,48^.

Notably, where indications of spatial learning and memory impairment were observed with Tg rats in the 6-month cohort, the 9-month rats had no such genotype effects on WRAM performance. While previous work has shown spatial navigation deficits on the MWM in 10–11 month old TgF344-AD rats^11^, it is possible that the 9-month old Tg rats within our study utilized a different spatial strategy to solve this task to compensate for potential hippocampal compromise. In solving tasks like the WRAM, rats rely on a hippocampal-dependent allocentric strategy, where robust extra-maze cues are often utilized to spatially navigate to the goal arm^49^. However, rats may also use a striatal-dependent egocentric strategy, which relies on body orientation as the reference frame, following a path of sequential right or left turns^49,50^. Prior work in aged rats and humans has demonstrated impaired allocentric navigation, where egocentric strategies remain largely successful^51,52^. In the case of AD, where hippocampal functioning may be compromised due to severe neuropathology, prior research has found that AD patients show more severe deficits when forced to use an allocentric strategy^53,54^. Therefore, it is possible that Tg rats at the 9-month timepoint shift from an allocentric strategy to an egocentric strategy due to significant neuropathological development. Future research should further investigate this potentially critical timepoint in the TgF344-AD model through examining their ability to solve a spatial navigation task when forced to only use an allocentric strategy, and whether this correlates with some critical timepoint in the trajectory of hippocampal neuropathology.

At the 12-month timepoint, significant working memory impairments were observed in TgF344-AD rats relative to WT controls on the WRAM during the Asymptotic Phase of testing. This finding corroborates prior literature, where deficits in spatial learning and memory have been observed at 10–11 months of age on the MWM^11^ and 15 months of age on the Barnes maze^5^. Further, the trial-specific genotype effects for WMI errors at the 12-month timepoint indicate that rats did not differ in spatial working memory performance until the highest working memory load, where TgF344-AD rats had difficulty handling the significant strain on working memory processing as compared to WT rats. These findings indicate that, even at the 12-month timepoint, more nuanced behavioral assessments are necessary to gain insight into genotype-induced behavioral effects. Further work should be done to elucidate specific, fine-tuned cognitive domains to create a comprehensive behavioral profile which could provide further insight into key trajectories earlier in disease progression.

In contrast to the effects observed on the working and reference memory-dependent WRAM, results on the MWM indicated reference memory impairments for TgF344-AD rats relative to WT rats at every age-point assessed, that is at 6, 9, and 12 months old. Our noted impairment on the MWM is consistent with prior work^11^ showing significant spatial reference memory impairment on the hidden platform version of the MWM by 10–11 months of age in TgF344-AD rats compared to WT counterparts, with Tg-related impairments demonstrated in distance to the platform as well as multiple measures of qualitative swim segmentation outcomes including overall search trajectories and platform-direct swim paths. Markedly, in the 10–11 month-old cohort, female Tg rats demonstrated a lower proportion of direct trajectories to the hidden platform relative to all other groups including male Tg rats, with indications of similar effects in Tg females at the earlier ages as well^11^. The current findings highlight the critical need to evaluate sex as a variable when both sexes are tested in preclinical research concerning aging, AD, and AD-related dementias; collapsing across sexes without accounting for potential sex-related effects can lead to misleading interpretations. We also note here that MWM outcomes are different from the pattern of effects observed for RM outcomes on the WRAM. This coincides with several studies from our laboratory indicating that assessing reference memory in conjunction with working memory differs from assessments of one memory type alone, effects seen in both young and aged females^35,40,55^.

The physiological measures further characterize this model by providing information on genotypic differences beyond that of cognition across 3 age-points. Weekly measurements of body weight demonstrated that, at each age, female Tg rats weighed more than their female WT counterparts, suggesting that this TgF344-AD genetic profile may have a significant impact on key physiological factors such as satiety, exercise, and metabolism. This finding within the current AD rat model may seem unexpected, as weight loss is consistently observed in patients with AD^56^. However, in rodent models of AD, body weight outcomes have been shown to be impacted by sex, age, and type of AD model^22,57–61^. In fact, one study demonstrated that 3×Tg mice weighed more than their WT counterparts in young adulthood; however, by 12 months of age the Tg mice weighed less than WT mice even though they consumed more food, supporting the age-related development of a hypermetabolic profile^58^.

Like body weight, heart weights also indicate a unique physiological profile for the TgF344-AD model. We found that the 9-month timepoint was distinct, with female Tg hearts weighing more than female WT hearts. However, when the ratio between heart weight and body weight was considered, it was revealed that, relative to body weight, Tg hearts weighed less than their WT counterparts distinctly at this 9-month timepoint. Further work assessing the trajectory of metabolic and cardiovascular changes across age in the TgF344-AD model will clarify how other organ systems interact with AD pathology. Finally, reproductive system evaluations collected throughout the study (vaginal cytology) and at euthanasia (uterine weight) suggest that reproductive aging may differ across the Tg and WT rats. This is an exciting area for further research, as relationships between ovarian hormone changes and AD-like behavior and neuropathology with aging have been established preclinically^62^ and clinically, with work showing that abrupt loss of circulating ovarian hormones due to oophorectomy increases risk for dementia decades later in life^63^.

Western blot results of relative A$\beta$_1–42_ expression provided insight into the progression of AD-like pathology, with increasing levels of A$\beta$_1–42_ expression at each age in brain areas critical to learning and memory functioning. Our current work fits with the prior literature surrounding the TgF344-AD model, such that amyloid deposition has been detected at 6 months of age in the hippocampus^5^. The fact that pathology is present before robust behavioral deficits in the TgF344-AD rat model is profoundly useful for clinical translation, as this more closely mirrors the clinical trajectory of AD^2^. Amyloid beta was the pathological feature chosen for the present study in order to corroborate previous work done by Cohen and colleagues^5^ as well as to focus the resources available for this study on this brain measurement and the primary behavioral outcomes. Future research should characterize the other neuropathological facets of this AD model, such as the presence of tau pathology resulting in neurofibrillary tangles, gliosis, and neuronal loss, expanding upon the initial work of Cohen et al.^5^, to determine its effectiveness at each age-point in modeling the progression of AD. Finally, the evaluation of relative A$\beta$_1–42_ expression in the brain allowed for us to determine whether relationships existed between pathology and behavioral or physiological measures for each age. There were no significant correlations between behavioral outcomes and A$\beta$_1–42_ expression, and this lack of relationship seems to parallel clinical AD research. Indeed, while decades of research have been dedicated to the study of senile plaques as the characteristic hallmark of AD, there is a general lack of correlation between disease manifestation and overall amyloid pathology^64,65^. Recent research indicates that the clinical features of AD more closely align with tauopathy as well as soluble Aβ oligomers, rather than the insoluble fibrillar amyloid within dense-core plaques, as neuronal and synaptic loss commonly parallel these pathological trajectories^66,67^. By further evaluating the pathology of the TgF344-AD rat model in the future, additional work can determine whether relationships exist between other neuropathological markers of disease progression and behavior. The current investigations did, however, result in the discovery of a significant negative correlation between A$\beta$_1–42_ expression in the FC and body weight for female 9-month Tg rats, indicating that increased AD-like pathology was associated with decreased body weight. This finding fits with those of the clinical literature, where researchers have found that greater weight loss is associated with further progression of AD^68^. This provides greater context to the main effect of body weight also mentioned earlier: while Tg rats weighed more than their WT counterparts at this 9-month timepoint, greater body weight was associated with lower A$\beta$_1–42_ expression. Future work should further investigate this 9-month age as a putative critical period within this model, with an eye toward its potential for therapeutic intervention and cross-system interdisciplinary interpretations.

In summary, in the herein interdisciplinary experiment assessing select behavioral, physiological, and pathological outcomes of the TgF344-AD rat model across multiple key ages, we chose to focus on females as they are disproportionately affected by AD in the clinic, and they are understudied preclinically with most experimental approaches evaluating males solely or combining across the sexes, with reference to AD-related questions. Specifically, our data provide insights into the behavioral, physiological, and pathological profiles of female TgF344-AD rats across 6–12 months of age. Our battery of spatial learning and memory tasks found unique outcomes indicating impairment as early as 6 months of age, with profound working and reference memory impairment by 12 months. The lack of significant spatial working memory impairment at 9 months of age, in conjunction with a unique correlation between physiological and brain outcomes at this age-point, suggest that this may be a critical time for female TgF344-AD rats. Additionally, this study highlights the importance of considering the broader physiological consequences that coincide with behavioral deficits and pathology across this AD-like trajectory, with unique findings for body weight, heart weights, and uterine weight. Such systems-wide approaches in the clinic with AD diagnosis and treatment could improve outcomes. Our results highlight the importance of the initiatives driving the field toward a greater focus on cross-systems multidisciplinary research, where an emphasis has been placed on personalized medicine^69,70^ and the identification of biomarkers to assist in detecting early AD progression in vulnerable patients^71^.

## Supplementary Information

Supplementary Legends.

Supplementary Information 2.

Supplementary Information 3.

Supplementary Information 4.

## Data Availability

Data from the current experiment are available upon reasonable request to the corresponding author, Heather A. Bimonte-Nelson (Bimonte.nelson@asu.edu).
