# Supplementary material for: Task-dependent learning and memory deficits in the TgF344-AD rat model of Alzheimer’s disease: three key timepoints through middle-age in females
Source: Sci Rep. 2022 Aug 26;12:14596. doi: 10.1038/s41598-022-18415-1 (PMC9418316; doi:10.1038/s41598-022-18415-1)
Supplement: Supplementary file 1 — Supplementary Legends. [file 41598_2022_18415_MOESM1_ESM.docx]

**Supplemental Figure 1. Representative PCR results for genotypic designation of WT or Tg rats.** Image of a complete DNA gel, where red boxes highlight a lower band (400bp) that corresponds to the presence of the APP transgene, indicating a genotype of Tg, and a higher band (750bp) that corresponds to the presence of an internal control gene, indicating a WT rat.

**Supplemental Figure 2. Performance on the VP Task demonstrates consistent visual and motor acuity across ages and genotypes.** Latency (in seconds) across Trials 1-6 on the VP task, where at (a) 6 months, (b) 9 months, and (c) 12 months of age, Tg and WT rats did not differ in performing the procedural components of a water escape task. **p* < 0.05, ****p* < 0.001.

**Supplemental Figure 3.** **Representative images of full-length Western blots evaluating A**$\boldsymbol{\beta}$**_1-42_ and beta-actin expression for each brain region.** Images of full-length Western blot scans, where red boxes correspond with the cropped bands represented in Figure 8. For frontal cortex representative bands, see Figures 8A (beta-amyloid 1-42 bands) and B (beta-actin bands). For representative bands from the dorsal hippocampus representative bands, see Figures 8C (beta-amyloid 1-42 bands) and D (beta-actin bands). For entorhinal cortex representative bands, see Figures 8E (beta-amyloid 1-42 bands) and F (beta-actin bands). Each blot contains bands from a given experimental sample or +/- control that correspond to relative A$\beta$_1-42_ expression at approximately 4kDa and relative beta-actin expression at approximately 45kDa.
