# Supplementary figures and images for: Task-dependent learning and memory deficits in the TgF344-AD rat model of Alzheimer’s disease: three key timepoints through middle-age in females
Source: Sci Rep. 2022 Aug 26;12:14596. doi: 10.1038/s41598-022-18415-1 (PMC9418316; doi:10.1038/s41598-022-18415-1)

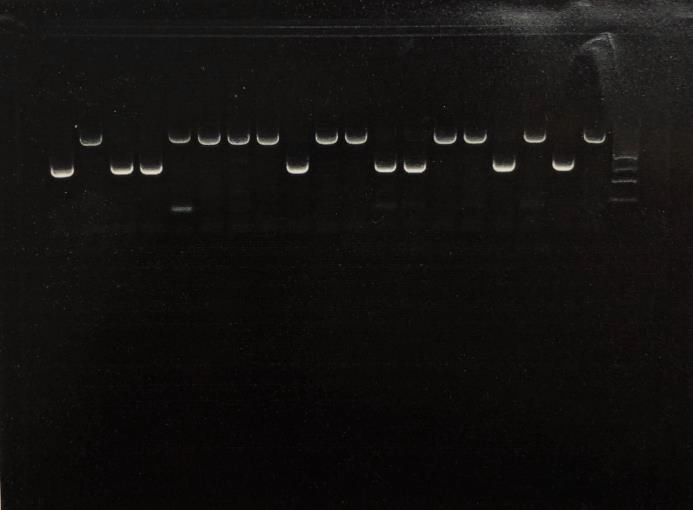


TG

WT

Supplement: Supplementary file 2 — Supplementary Information 2. [file 41598_2022_18415_MOESM2_ESM.docx]

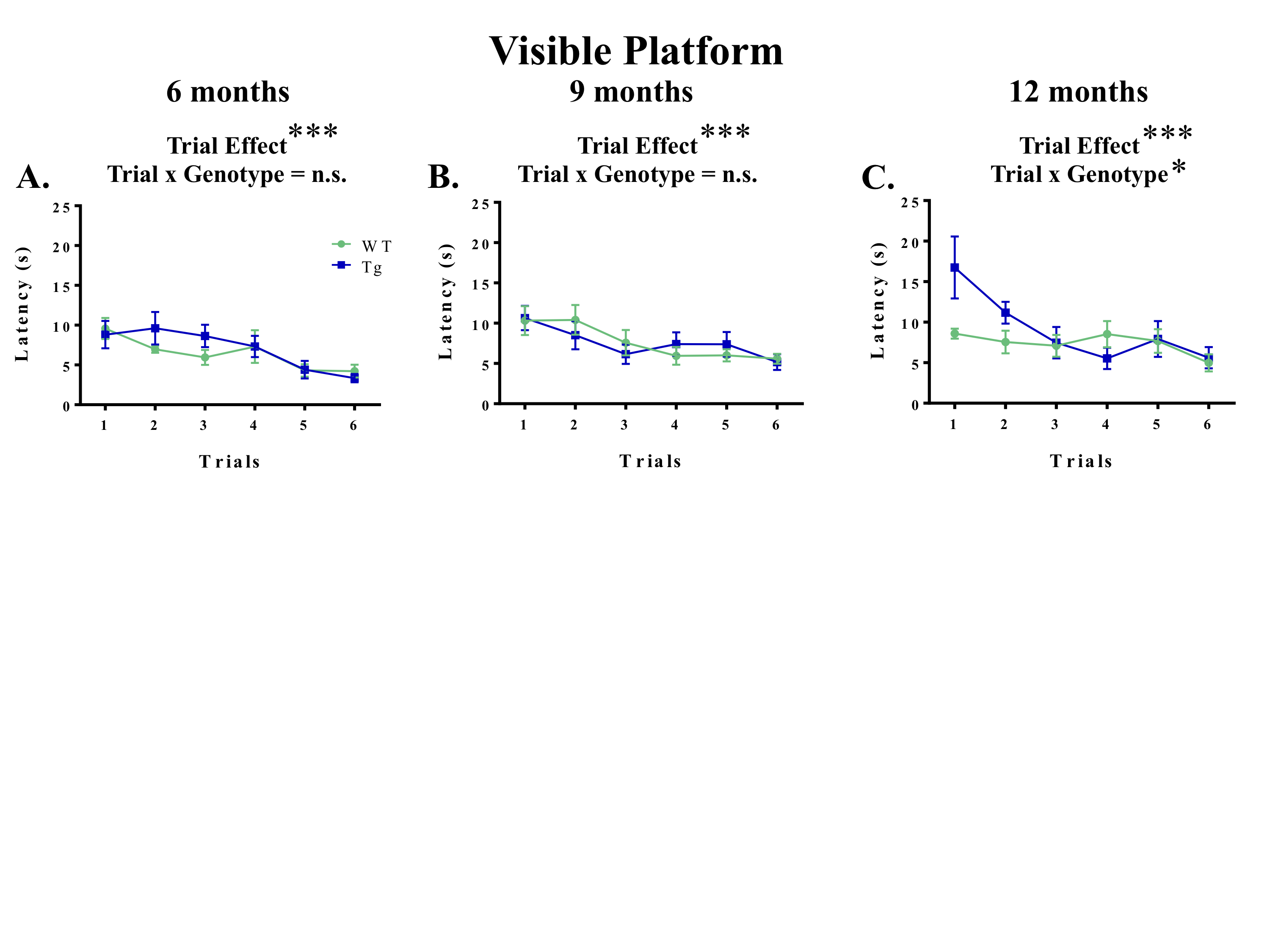

Supplement: Supplementary file 3 — Supplementary Information 3. [file 41598_2022_18415_MOESM3_ESM.tiff]

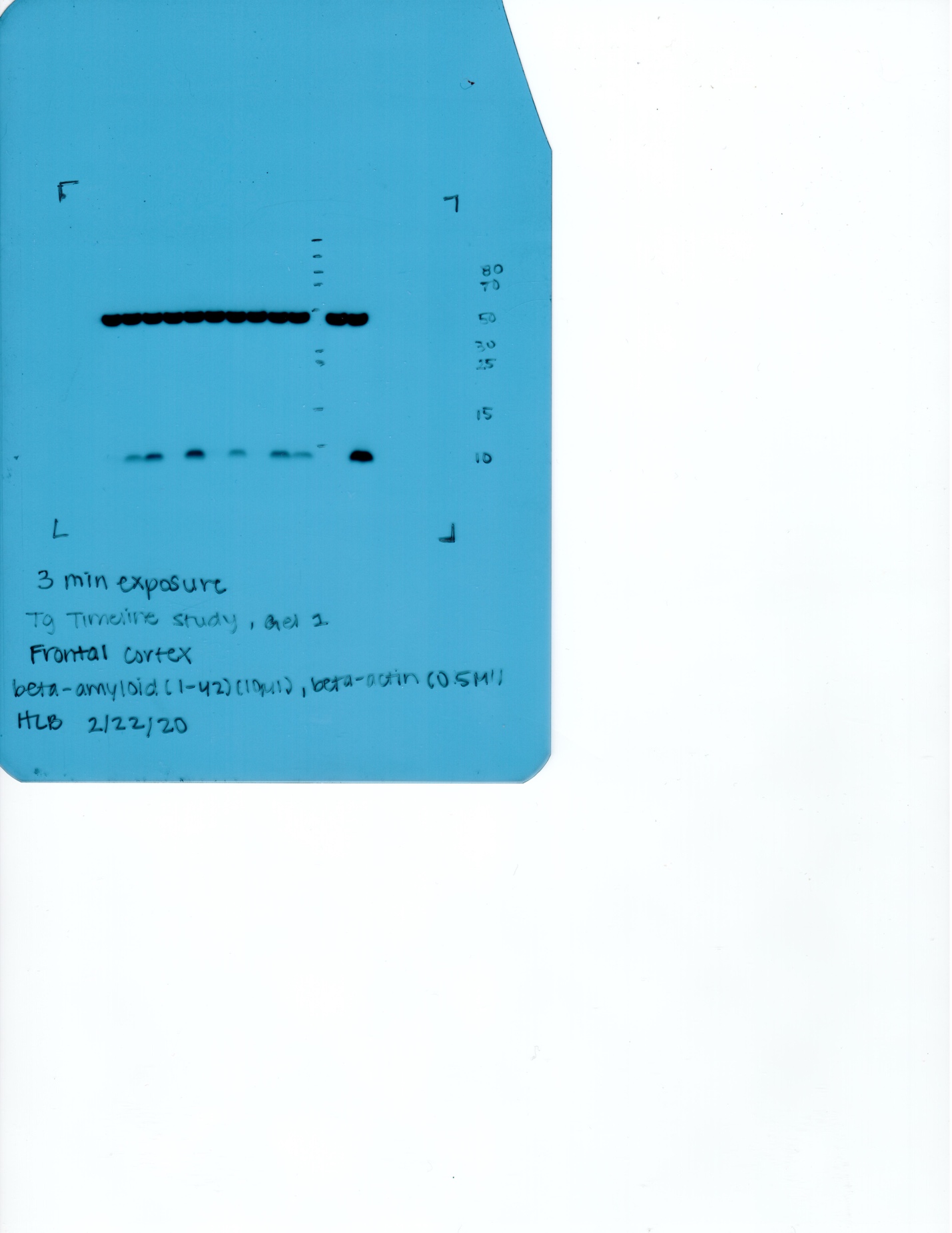


**A.**


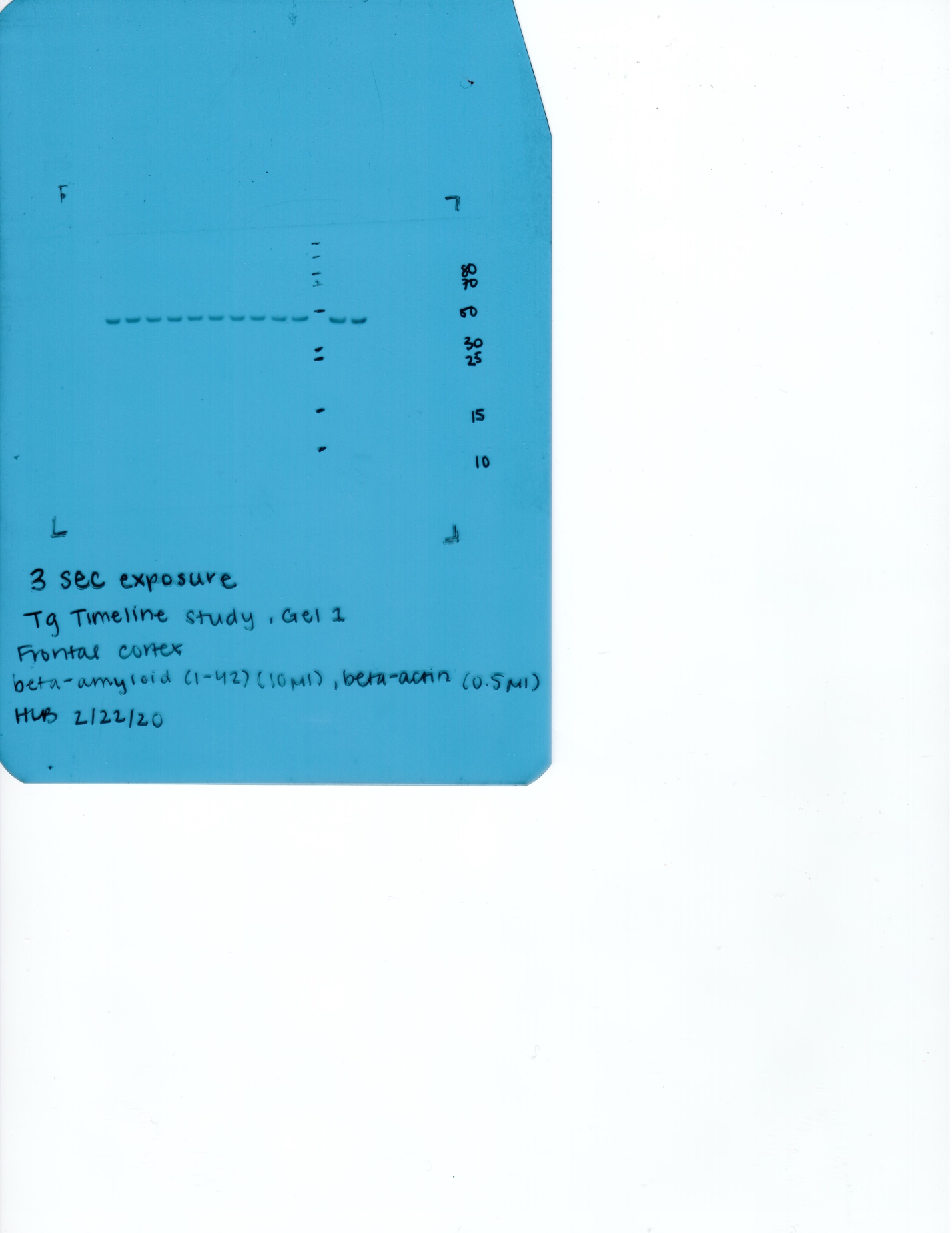


**B.**


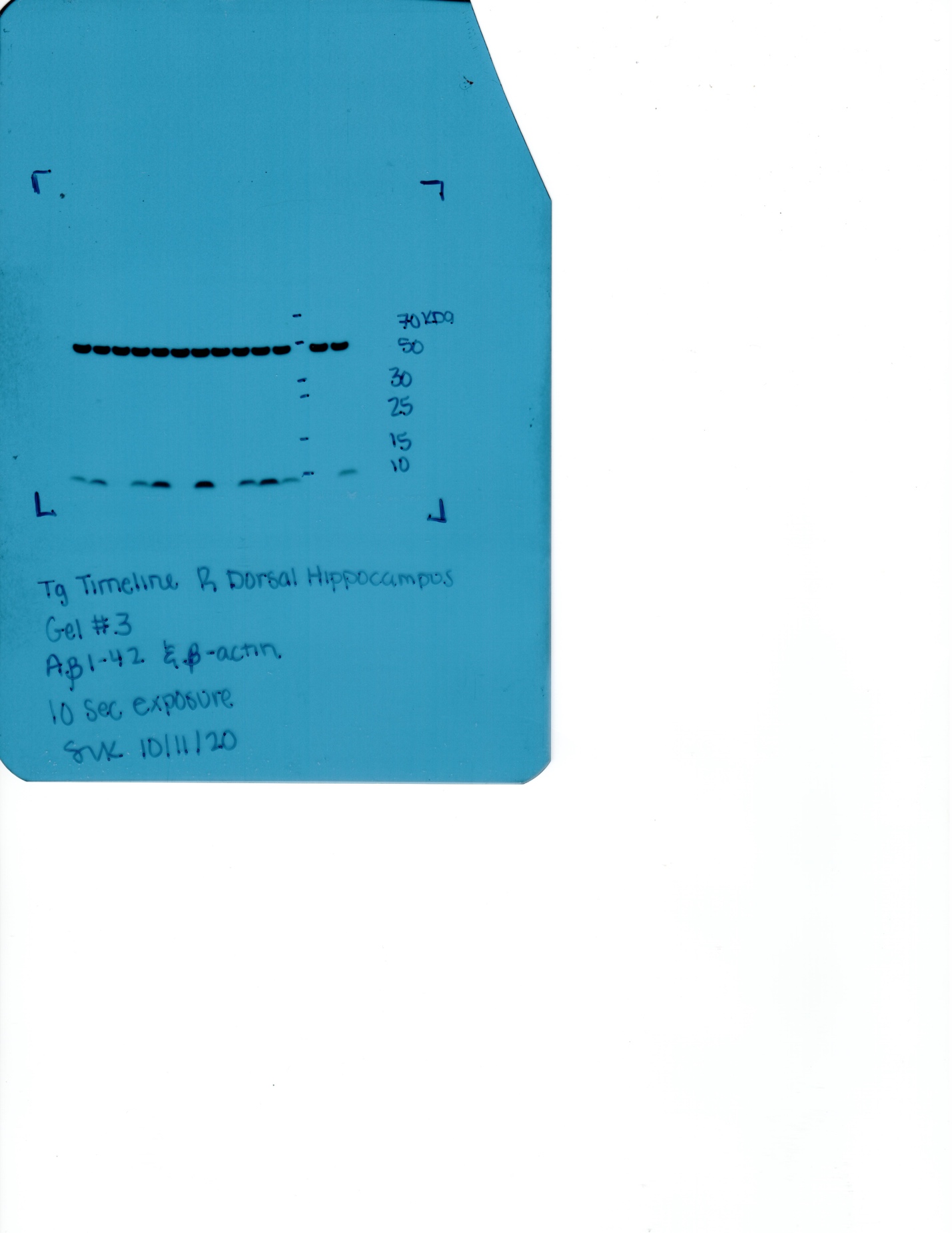


**C.**


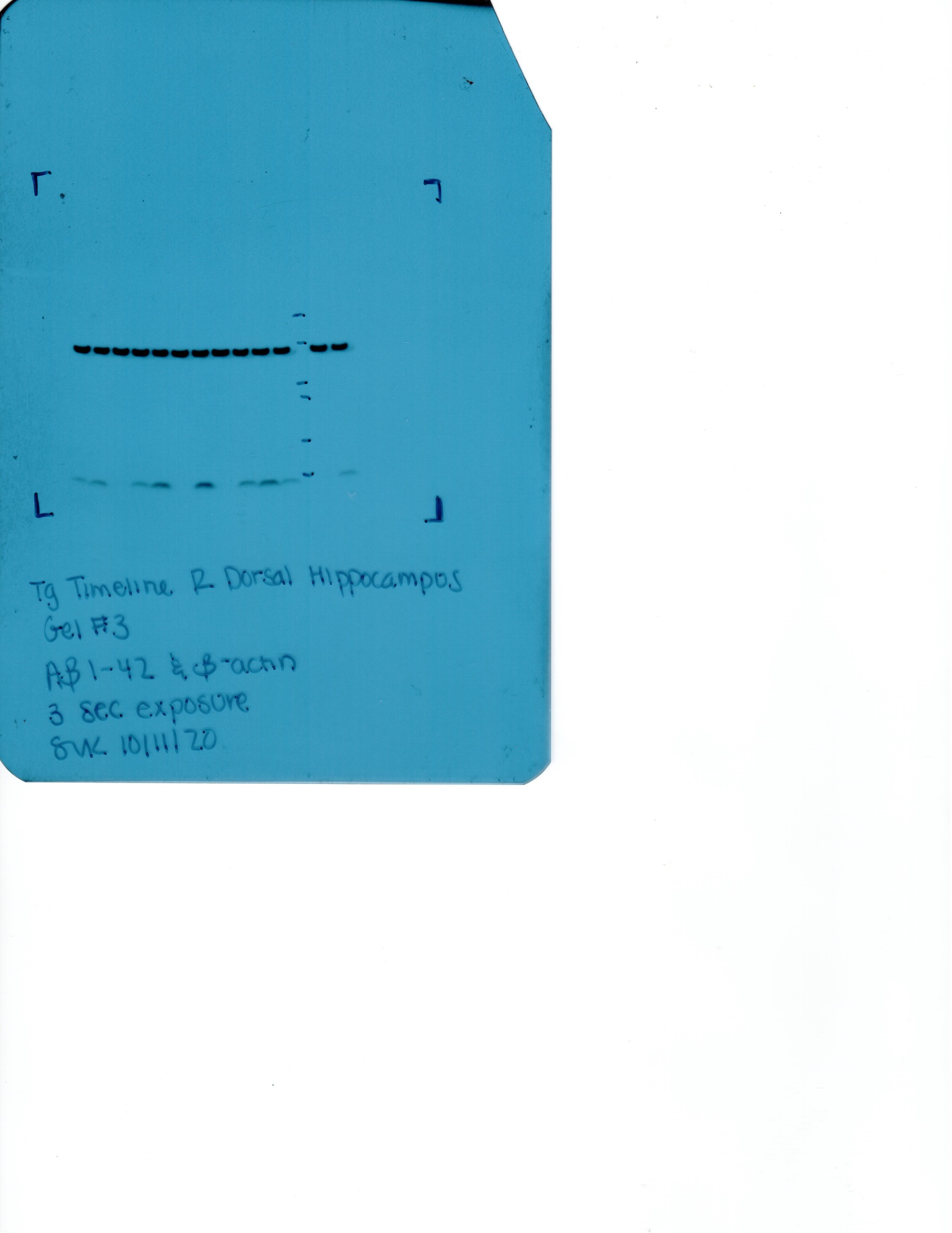


**D.**


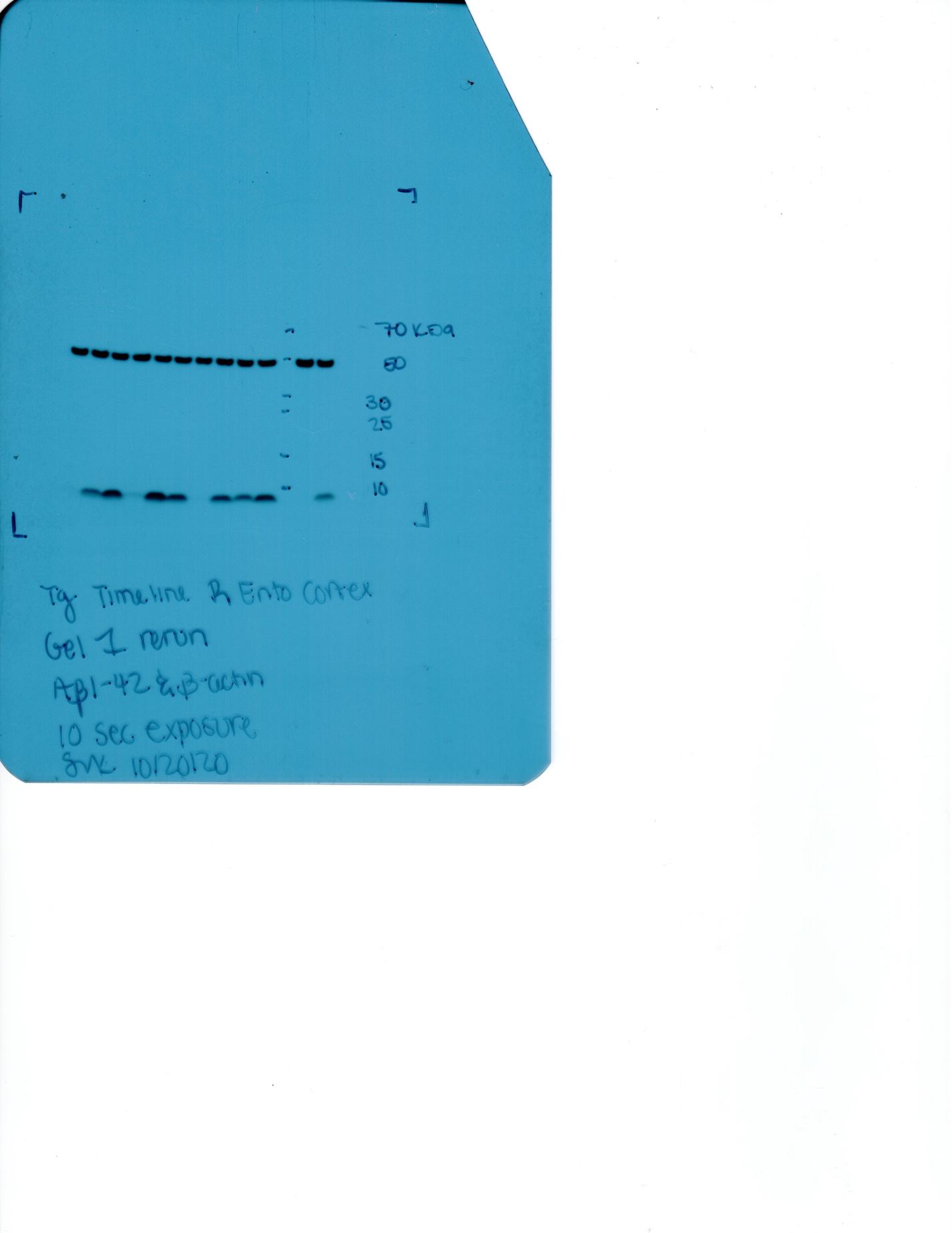


**E.**


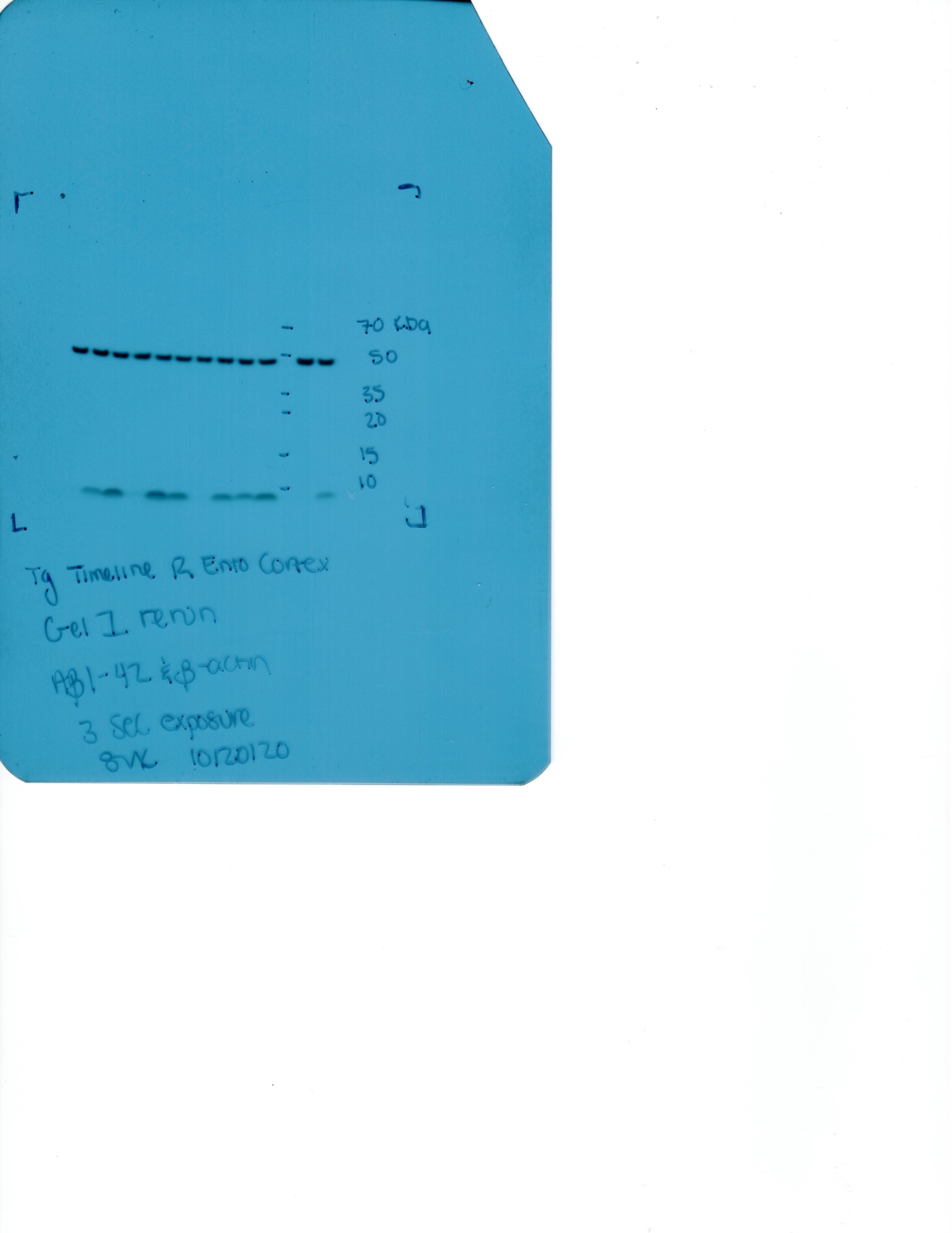


**F.**

Supplement: Supplementary file 4 — Supplementary Information 4. [file 41598_2022_18415_MOESM4_ESM.docx]
